# Inverted *J*–*V* Hysteresis in Perovskite Solar Cells: Insights from Photovoltaic Quantum Efficiency

**DOI:** 10.1021/acsenergylett.5c04035

**Published:** 2026-01-30

**Authors:** Miguel Torre Cachafeiro, Carys A. Worsley, Fuxiang Ji, Trystan M. Watson, Wolfgang Tress

**Affiliations:** † Institute of Computational Physics, 30944 Zurich University of Applied Sciences (ZHAW), 8400 Winterthur, Switzerland; ‡ Institut des Matériaux, École Polytechnique Fédérale de Lausanne (EPFL), 1015 Lausanne, Switzerland; § 7759 Swansea University, Bay Campus, Neath, Skewen SA18EN, Wales

## Abstract

The most typical hysteresis in the current density–voltage (*J*–*V*) curve of perovskite solar cells (PSCs) shows better performance in the backward (BW) than in the forward (FW) voltage scan (normal hysteresis). The opposite, where the FW scan yields higher photocurrent, is known as inverted hysteresis and is also frequently observed. Here, we examine PSCs exhibiting both normal and inverted hysteresis, depending on scan rate and preconditioning. Spectral changes in the external quantum efficiency (EQE) linked to ionic redistribution reveal that inverted hysteresis arises from blue-range photocurrent losses caused by enhanced recombination at the interfaces due to ionic accumulation. This trend is consistent across PSC architectures, as demonstrated for triple mesoscopic carbon-based (C-PSCs) and planar p-i-n devices. Combined with drift-diffusion simulations, the results show that ionic losses can be bidirectional, and the hysteresis direction depends on how the ionic distribution impacts charge collection efficiency.

## Graphical abstract

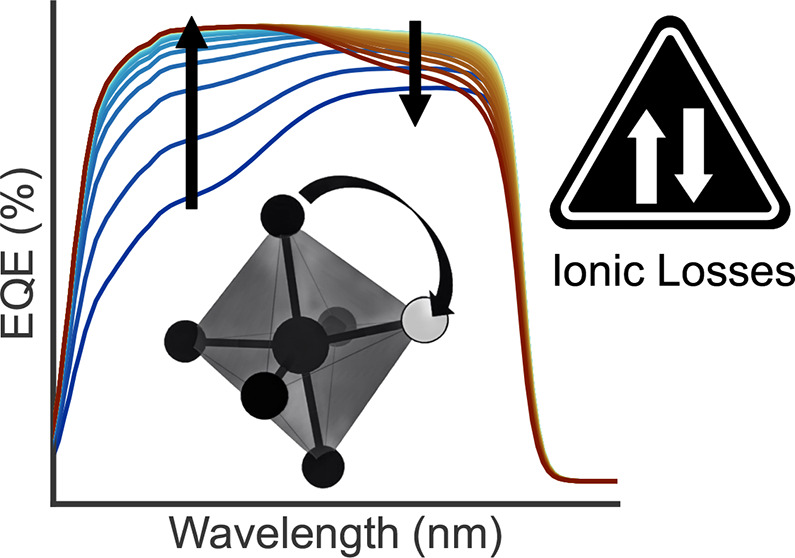

Perovskite solar cells (PSCs) may exhibit a scan rate-dependent hysteresis in current density–voltage (*J*–*V*) curves due to mobile ions.^1,2^ When “normal hysteresis” (NH) occurs, the backward (BW) scan yields higher photocurrent than the forward (FW) scan, which is affected by the bulk electric field inversion caused by the ionic space charge.^3,4^ Typically, near-*V*_OC_ preconditioning (and measuring fast before ions can respond) can prevent the charge collection efficiency loss due to the electric field screening effect of mobile ions.^5,6^ While the correct ionic loss could be measured relative to the precondition bias at which the ionic space charge vanishes,^7,8^ an important ambiguity remains; ionic redistribution can also lead to the opposite outcome, where prebiasing the device (in theory preventing ionic screening) results in lower current collection, as may be the case for “inverted hysteresis” (IH), where the FW scan yields higher current than the BW one.^9–12^ Since *J*–*V* hysteresis can manifest in either direction (normal or inverted), ionic losses in PSCs must effectively be a “two-way street”, which suggests that the current understanding of ion-induced losses in PSCs is still incomplete.

Understanding the direction of hysteresis, depending on the precondition and scan rate used, is crucial to clarify how mobile ions affect recombination in different devices. Although most high-efficiency PSCs nowadays show minimal initial hysteresis at the reported scan rates,^13,14^ this does not mean they are free from ion migration.^15^ In fact, a PSC does not necessarily need to be hysteresis-free to achieve a high efficiency, since the bulk field inversion caused by the delayed ionic response^4^ is something that will not happen under stabilized maximum power point conditions. This means that some hysteresis can be present even in the radiative limit, as long as there are mobile ions. With aging, the deterioration of electronic properties can introduce significant hysteresis, making its understanding still highly relevant for PSC development.^16^ For the case of IH, previous interpretations remain varied,^12,17–20^ missing a simple and unified understanding. Here, we demonstrate that IH may arise when ionic accumulation under a positive precondition bias causes an enhancement of recombination at the interfaces, which outweighs the effect of reduced bulk field screening. Using photovoltaic external quantum efficiency (EQE) measurements and drift-diffusion simulations, we show how this mechanism applies across device architectures, comparing triple mesoscopic C-PSCs and fully planar p-i-n devices.

## Triple Mesoscopic C-PSC

Figure 1 shows rate-dependent *J*–*V* curves for HTL-free C-PSCs with a triple mesoscopic structure, showing pronounced IH behavior following a 30 min *V*_OC_ precondition prior to a BW–FW scan (Figure 1a–b). A clear trend is observed: the fast “ion-freeze” scan^6^ gives the lowest *J*_SC_; IH dominates at an intermediate rate (50 mV s^–1^); and NH emerges when the scan rate is reduced further (1 mV s^–1^). In agreement with this trend, the *J*_SC_ transient from a sudden change from *V*_OC_ to short-circuit (Figure 1b) shows an initial increasing regime followed by a decreasing one later in time, explaining the rate-dependent hysteresis direction. Faster scans operate on the increasing *J*_SC_ regime, which tends to show higher current later in time during the FW scan (IH), thanks to the transient recovery process as ions redistribute at the lower applied voltages. Slower scans fall on the time scales of the decreasing *J*_SC_ regime, characteristic of losses due to bulk ionic screening (NH).^4^ Further discussion on the interplay between ionic distribution and dominant recombination mechanisms can be found in Supplementary Note 1 in the Supporting Information (SI).

**Figure 1:**
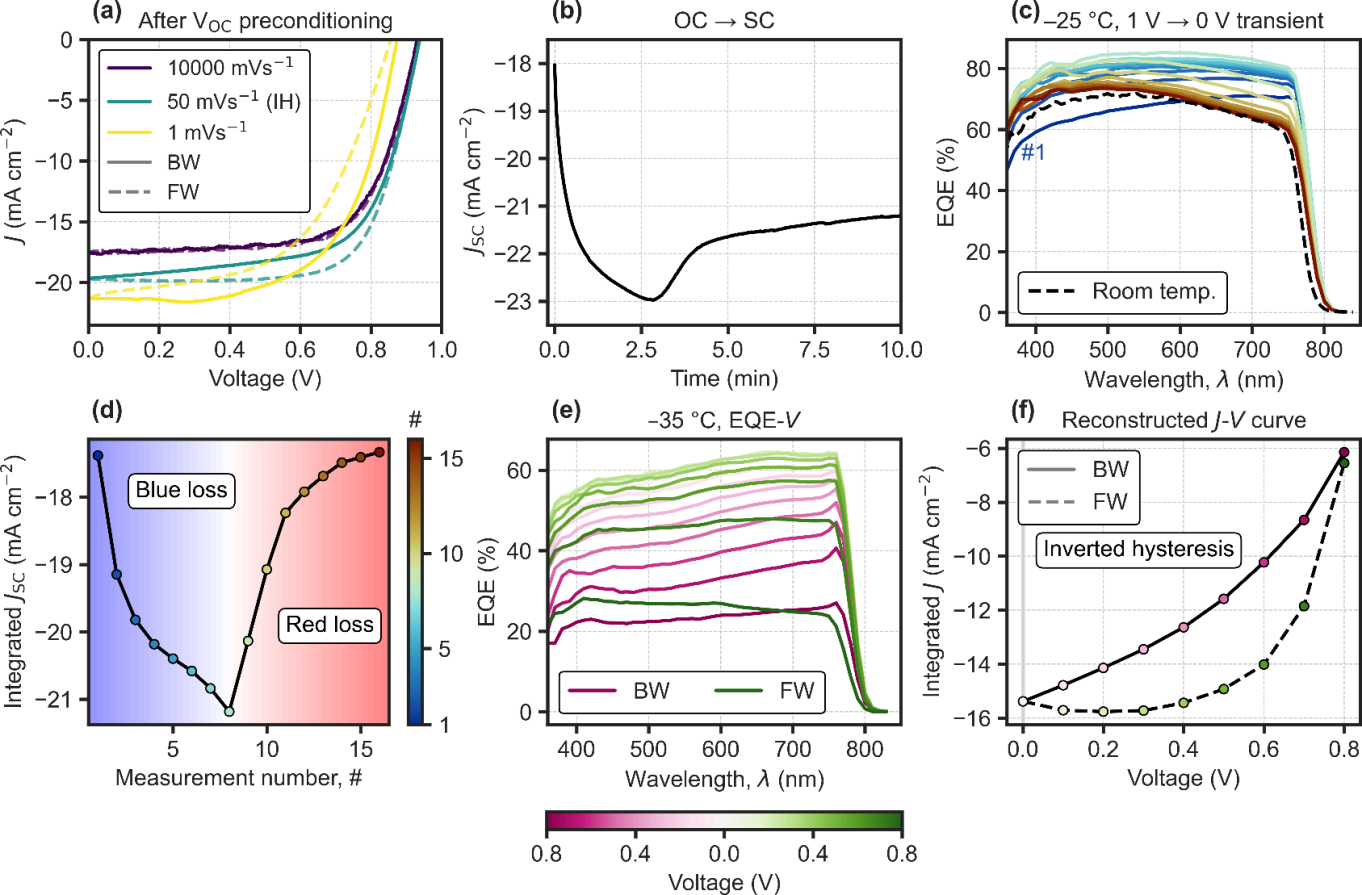
C-PSC. (a) *J*–*V* curves starting with the BW scan for varying scan rates; fast, medium and slow (10 000, 50 and 1 mV s^–1^, respectively). Measured under AM1.5G solar simulator after ∼30 min of preconditioning at *V*_OC_ under illumination. The medium-rate scan shows pronounced inverted hysteresis (IH). (b) *J*_SC_ transient after *V*_OC_ preconditioning. (c) Sequential EQE at short-circuit at low temperature (−25 °C), after 1 V preconditioning. (d) Integrated *J*_SC_ from (c) assuming AM1.5G spectrum. (e) Sequential voltage-dependent EQE at low temperature after 1 V preconditioning. (f) Reconstructed *J*–*V* curve from (e), assuming AM1.5G spectrum.

Spectral analysis of sequential EQE measurements at short-circuit after a 1 V precondition reveal the origin of the ion-induced current losses (Figure 1c–d). Initially after the precondition, losses occur predominantly in the blue spectral region. As ions redistribute toward their 0 V steady state, blue losses tend to decrease until a maximum EQE is reached. This is then followed by the increase of the characteristic red loss due to bulk ionic screening in these devices.^4,21^ To confirm that IH operates on the blue loss regime, voltage-dependent EQE was measured to reconstruct the *J*–*V* with spectral information. A 1 V precondition was applied before measuring EQE–*V* in the same order as the BW-FW voltage scan, using a temperature low enough to mimic an intermediate scan rate, due to the lowered ionic mobility (Figure 1e–f). As expected, IH originates from the transient recovery process of the blue loss as the applied voltage is lowered and ions redistribute, which results in higher current values later in time, during the FW scan.

To validate our interpretation, we performed 1D drift-diffusion simulations coupled with an optical model (parameters in SI Table S2). The model includes mobile cations (Figure 2a) (1 × 10^16^ cm^–3^) with a static anion countercharge, representing iodine vacancy-mediated transport.^22^ Key features include a deep TiO_2_ conduction band (0.2 eV offset)^23^ and a high ETL-perovskite interface recombination velocity (5 m s^–1^). The effect of ion migration as seen in the experimental EQE transient in Figure 1c is modeled using precondition voltages, which define the different ion distributions as shown in Figure 2a (see Supplementary Note 1). The simulated EQE spectra (Figure 2b) reproduce the experimental trends: high positive preconditions cause blue losses due to cation accumulation at the front side of the device, while below the “ion-free” voltage, at which the net ionic charge reaches a minimum,^8^ red losses tend to increase with bulk electric field screening,^4^ now due to cation depletion at the front. The model captures both spectral dependencies on the ionic distribution, highlighting how blue losses and inverted hysteresis arise from a high sensitivity of the recombination to ionic accumulation above the “ion-free” voltage, where the polarity of the ionic space charge layers inverts (Figure 2a). Analysis of the driving force for charges, the quasi-Fermi level gradients (Figure S6), shows how holes photogenerated near the front of the device tend to be driven toward the wrong contact under high positive preconditions of the ions, enhancing recombination at the front electron-collecting contact. While the *J*_SC_ tends at some point to decrease with a high voltage (precondition) ionic distribution, the *V*_OC_ value shows in this case the opposite trend as it benefits from the ionic screening at OC under illumination (Figure 2c). Figure 2d–e shows how the spectrally resolved recombination currents at short-circuit evolve with increasing preconditioning voltage, highlighting the shifting balance between blue (interface) and red (bulk) losses. In contrast, at OC, a high positive precondition helps to suppress interfacial recombination as seen in Figure 2f, due to less or no inversion of the bulk electric field at OC (Figures S7–S8) and resulting in a higher *V*_OC_, as observed experimentally (see Supplementary Note 2 in the SI). For C-PSCs, the presence of IH seems to depend on the properties of the m-TiO_2_ layer,^24^ as also discussed in Supplementary Note 2 (SI).

**Figure 2:**
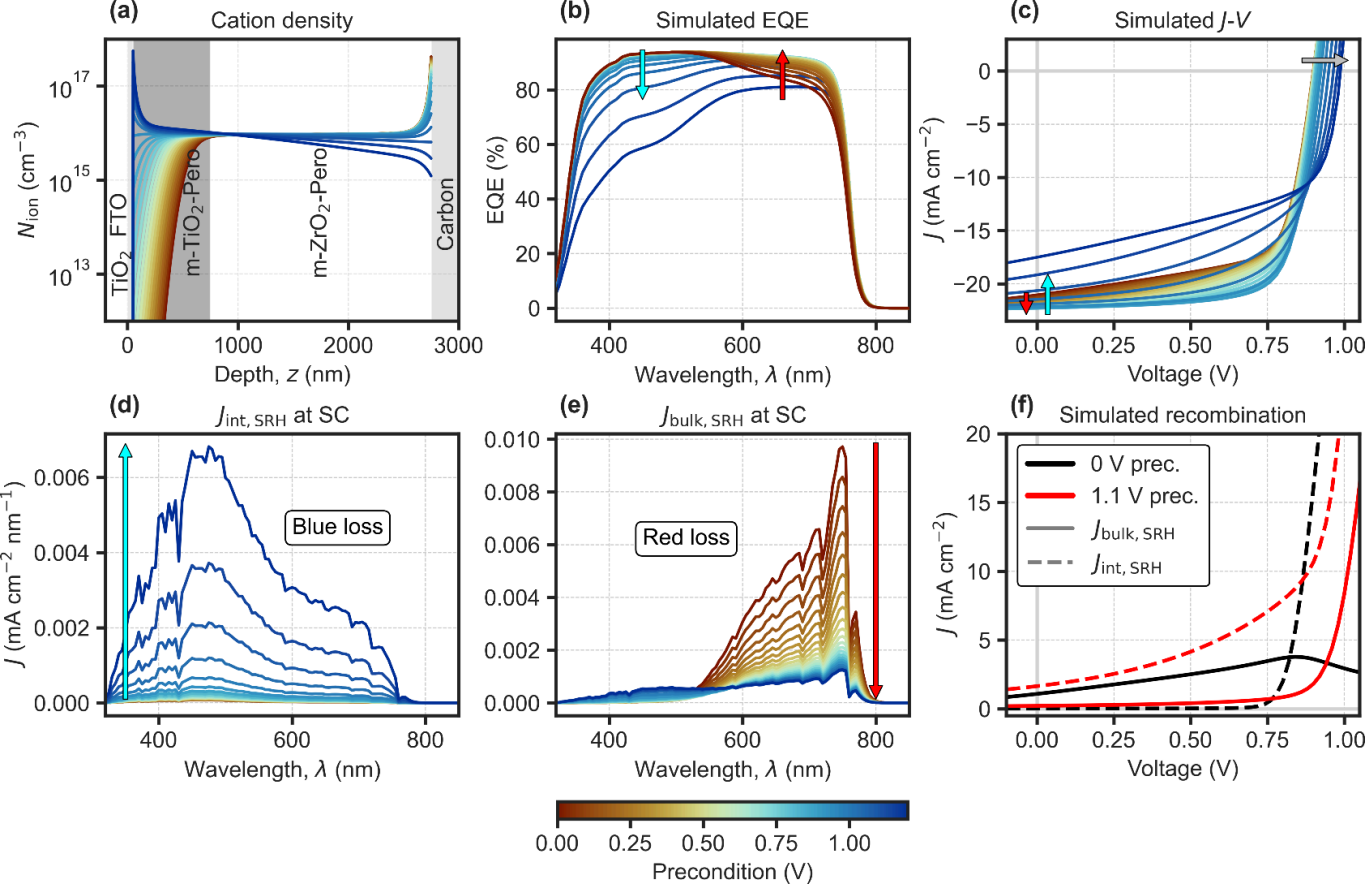
C-PSC. Optical and drift-diffusion simulations for different precondition voltages for ions. (a) Cation density profiles. (b) EQE spectra at short-circuit, where the arrows show the trend with increasing precondition voltage. (c) *J*–*V* curves. (d) Front interface recombination current (blue loss) and (e) bulk SRH recombination current (red loss) at short-circuit (SC), with AM1.5G spectrum. (f) Recombination currents under illumination for the *J*–*V* curves in (c), showing the 0 and 1.1 V preconditions.

## Fully Planar p-i-n PSC

To confirm whether the observed trends can be generalized across device architectures, we examined planar p-i-n PSCs (Figure 3) where hole extraction now occurs at the front side instead, using a self-assembled monolayer (SAM).^25^ We note that the device was subjected to 300 h of maximum power point tracking under illumination to show pronounced ion-dependent losses, but that some level of IH is also often present in fresh devices (Figure S10). Again depending on the scan rate, mixed hysteresis directions could be observed, now with IH appearing at the slower rates (Figure 3a). As shown in Figure 3b, the *J*_SC_ transient upon switching from open-circuit shows also in this case an increasing regime, this time in the slower time scales, consistent with the IH observed for the slowest scan. Sequential EQE measurements after cooling to −25 °C under 1 V and switching to short-circuit (Figure 3c), reveal how the slow *J*_SC_ increase originates from the transient recovery of blue losses after the preconditioning (1 V). The spectral changes observed confirm the enhanced front-side recombination due to preconditioning at high forward bias (Figure 3d), also for PSCs with an inverted p-i-n architecture. Simulations for the p-i-n structure (parameters in SI Table S3) reproduce the experimental observations, showing IH from a 1 V precondition due to enhanced interface recombination under positive voltage preconditions (Figure 3e). The analysis of energy band diagrams and quasi-Fermi levels (at short-circuit) confirm how, in this case the depletion of cations at the HTL side due to the positive voltage precondition, tends to drive electrons wrongly toward the front hole-collecting contact where they recombine (Figure S11). The forward bias precondition is significantly more detrimental than the equilibrated 0 V distribution in this case, since the bulk electric field screening of ions does not induce significant EQE losses. As a result, ionic relaxation leads to the transient recovery of blue losses responsible for IH, as can be seen in the simulated EQE–*V* map in Figure 3f, for the *J*–*V* curve with IH in Figure 3e. At low voltages, the blue loss tends to recover, meaning the collection efficiency increases over time as ions redistribute from the precondition, resulting in higher current during the FW scan (measured later in time).

**Figure 3:**
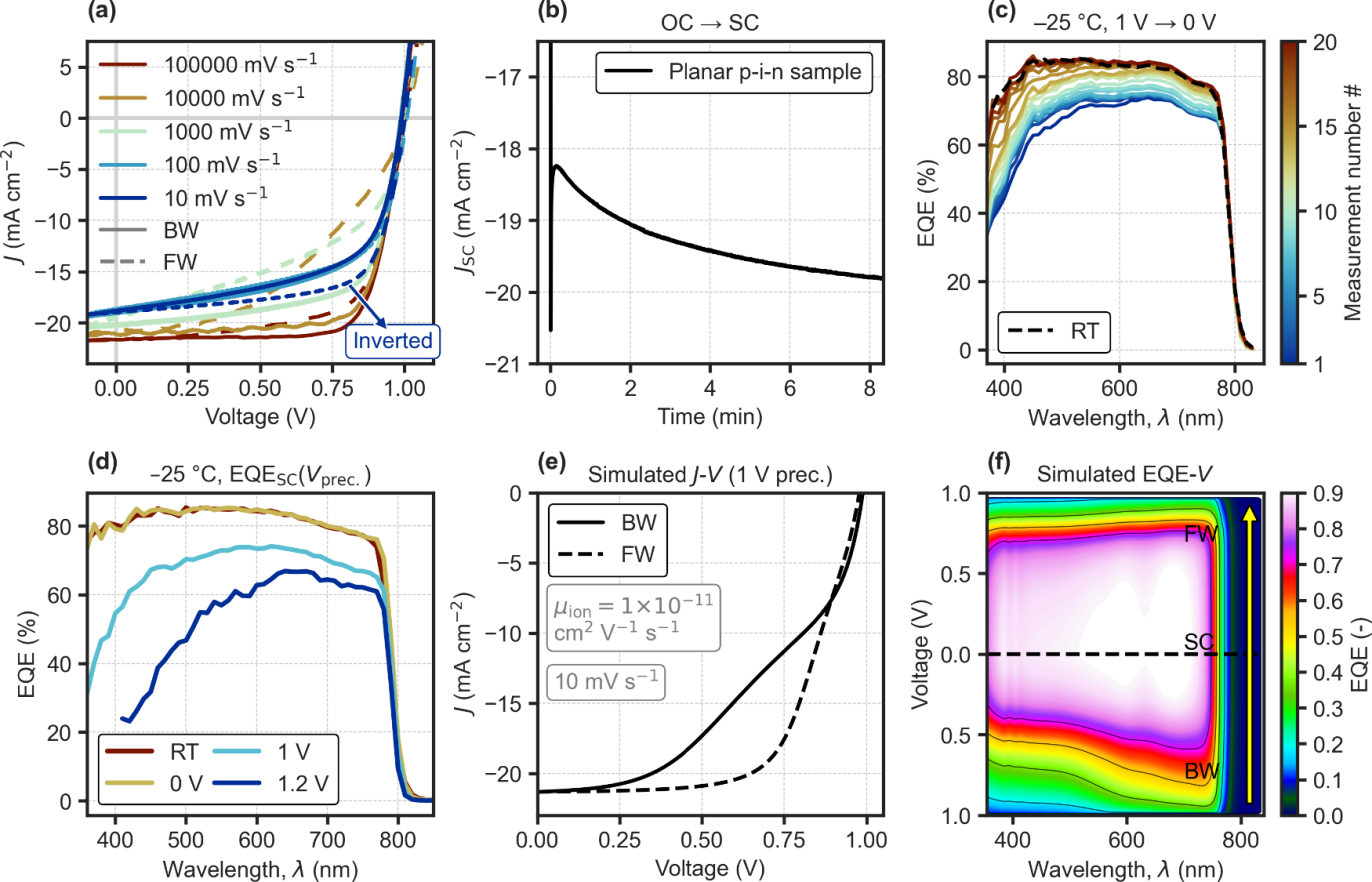
Degraded p-i-n PSC. (a) Rate-dependent *J*–*V* curves starting with the BW scan, measured under an AM1.5G solar simulator, from a *V*_OC_ precondition. (b) Transient *J*_SC_ upon switching from open-circuit (OC) to short-circuit (SC). (c) Sequential EQE measurements at short-circuit at low temperature (−25 °C), from a precondition around *V*_OC_ (1 V), applied while cooling down. (d) EQE at short-circuit at low temperature (−25 °C) after cooling down at different precondition voltages. (e) Simulated transient *J*–*V* curves from a precondition at 1 V, for a p-i-n device model (details in the SI), showing IH. (f) Computed evolution of EQE during the transient voltage scan in (e), shown as a color map.

## Interpreting Hysteresis Directions

The insights provided by spectrally resolved EQE measurements across device architectures establish a comprehensive framework to understand *J*–*V* hysteresis directions (Table 1). The hysteresis direction is dominated by whether the ionic distribution for the 0 V condition, where they screen the bulk electric field (Figure 4a), or the one for a high positive bias above the “ion-free” voltage (Figure 4b), result in more or less current losses. This depends on the carrier lifetimes and specific recombination pathways available, which determine the efficiency of diffusion-dominated transport as a result of reduced or inverted electric fields. In this sense, IH is a result of inefficient carrier transport near interfaces outweighing any positive effects from preventing bulk field screening under a positive bias precondition, as illustrated by the collection efficiency (η_coll_) profiles in Figure 4. The occurrence of IH can thus be interpreted as recombination in a device (under extraction conditions) being highly sensitive to the polarity inversion of ionic space charge layers around *V*_OC_, where this inversion occurs when the *V*_OC_ is higher than the “ion-free” voltage. The resulting blue loss (as seen in the EQE) may tend to slowly recover as the applied voltage is lowered, leading to IH when the BW scan is measured first. For comparison, n-i-p devices with NH only show increased EQE across all wavelengths with positive precondition voltages, though with a consistently lower enhancement in the blue spectral range (Figures S13–S14). This highlights how such devices are not as sensitive to this type of ionic accumulation, since the beneficial effect of preventing bulk electric field screening remains dominant. Reverse bias preconditioning (*V*_pre_ < 0) can also produce inverted hysteresis,^10^ in this case via bulk field inversion, causing dramatic red losses that tend to recover slowly (Supplementary Note 3 in the SI), providing another route to IH behavior, again due to the dominance of a background recovery process, characterized by an increasing *J*_SC_ transient upon a switch from the precondition voltage (Figures S15–S16). Altogether, the observations presented here provide a unifying picture to understand ion-distribution-dependent current losses and how different *J*–*V* hysteresis directions arise, based on how ionic space charge modulates the spatial collection efficiency. This insight can support PSC degradation analysis and targeted device optimization.

**Figure 4:**
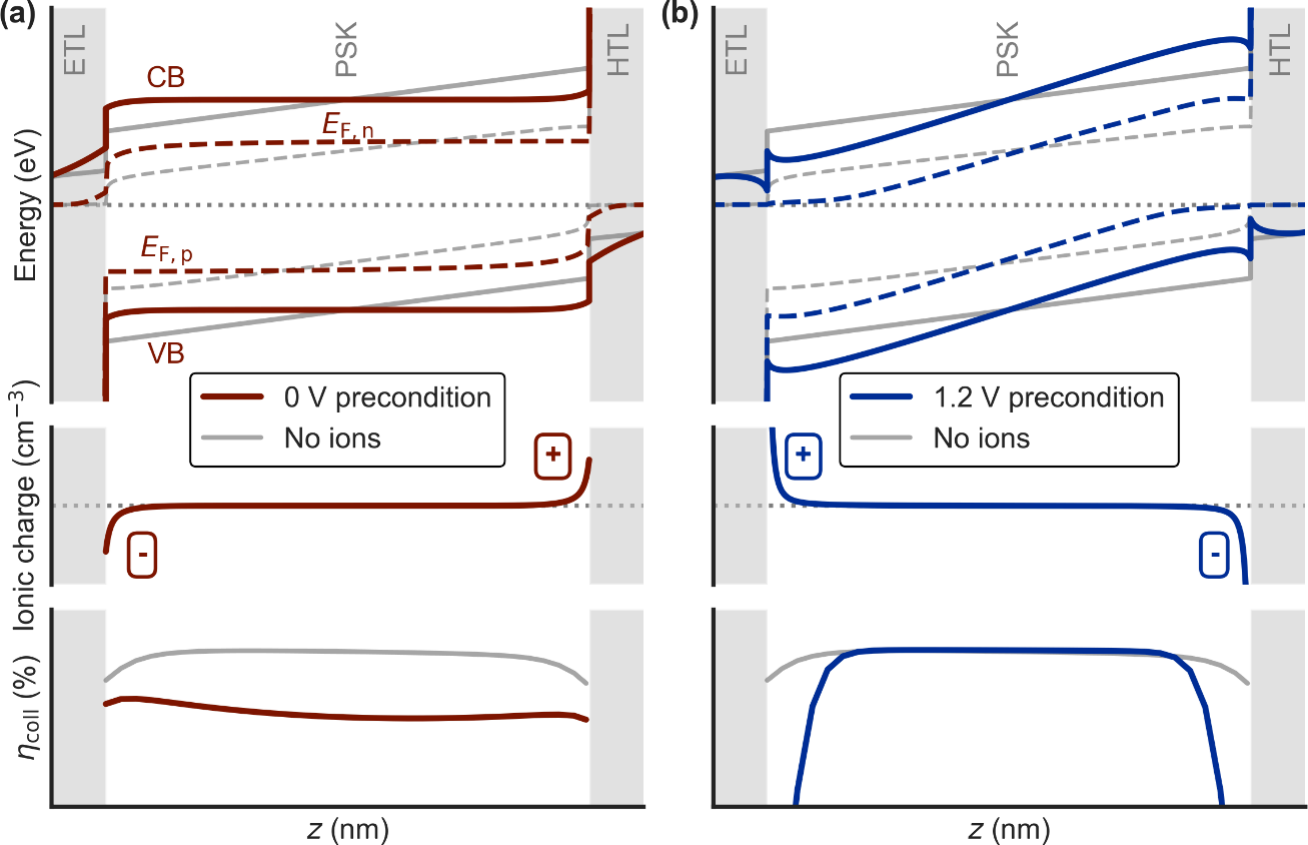
Schematic energy band diagrams, net ionic charge and collection efficiency (η_coll_) profiles for (a) 0 V and (b) 1.2 V preconditions, for a generic PSC at short-circuit (including both interfacial and bulk defect recombination).

**Table 1:** Hysteresis Direction Diagnosis, Assuming the Backward Scan Is Measured First

Precondition	Effect	*V*_pre_ → *J*_SC_	Hysteresis
*V*_pre_ ≈ *V*_OC_	Lower bulk or interface recombination thanks to lower bulk ionic screening.	*J*_SC_ *↓*	Normal
*V*_pre_ ≈ *V*_OC_	Recombination at or near interfaces increases, outweighs beneficial effect from lowered bulk ionic screening.	*J*_SC_ *↑*	Inverted/mixed
*V*_pre_ < 0	Higher recombination due to field inversion in the bulk.	*J*_SC_ *↑*	Inverted/mixed

## Methods

### Experimental Devices

The architecture of HTL-free triple mesoscopic C-PSC devices is FTO/TiO_2_/m-TiO_2_-AVA-MAPI/m-ZrO_2_-AVA-MAPI/Carbon, where MAPI stands for methylammonium lead triiodide (MAPbI_3_). The p-i-n device is composed of FTO/MeO-4PACZ/Perovskite/PZ2HI/​PCBM/BCP/Au, where the composition of the perovskite layer is (FA_0.95_MA_0.05_)_0.95_Cs_0.05_Pb­(I_0.95_Br_0.05_)_3_. All fabrication details are in the SI.

### Measurements

*J*–*V* curves were measured with BioLogic SP-300 potentiostat under AM1.5G solar simulator illumination. EQE was measured with a monochromator setup using lock-in detection (330 Hz) under different bias voltages and temperatures, controlled using a Linkam cryostat. Each EQE spectrum required ∼4 min measurement time.

### Simulations

Optical and drift-diffusion simulations were performed with Setfos 5.5 (Fluxim AG). The parameters used are detailed in SI Tables S2–S3.

## Supplementary Material



## Notes

Additional experimental data, discussions (Supplementary Notes 1–3), simulation parameter tables, simulated results, and device fabrication details (PDF)

## References

[1] Tress W., Marinova N., Moehl T., Zakeeruddin S. M., Nazeeruddin M. K., Grätzel M. (2015). Understanding
the rate-dependent
J–V hysteresis, slow time component, and aging in CH 3 NH 3
PbI 3 perovskite solar cells: the role of a compensated electric field. Energy Environ. Sci..

[2] Senocrate A., Moudrakovski I., Kim G. Y., Yang T.-Y., Gregori G., Grätzel M., Maier J. (2017). The nature of ion conduction in methylammonium
lead iodide: a multimethod approach. Angew.
Chem..

[3] Courtier N. E., Cave J. M., Foster J. M., Walker A. B., Richardson G. (2019). How transport
layer properties affect perovskite solar cell performance: insights
from a coupled charge transport/ion migration model. Energy Environ. Sci..

[4] Torre
Cachafeiro M. A., Narbey S., Ruhstaller B., Nüesch F., Tress W. (2025). Visualising ionic screening in perovskite
solar cells: a bumpy ride along the J–V curve. EES solar.

[5] Le
Corre V. M., Diekmann J., Pena-Camargo F., Thiesbrummel J., Tokmoldin N., Gutierrez-Partida E., Peters K. P., Perdigón-Toro L., Futscher M. H., Lang F. (2022). Quantification of efficiency losses due to mobile ions
in perovskite solar cells via fast hysteresis measurements. Solar RRL.

[6] HBalaguera E., Marinelli Pra F. J., Das C., Torresani L., Bisquert J., Saliba M. (2025). ‘Ion-freeze’ efficiency
in perovskite solar cells: time scales for ion immobilization. EES solar.

[7] Hart L. J., Angus F. J., Li Y., Khaleed A., Calado P., Durrant J. R., Djurišić A. B., Docampo P., Barnes P. R. (2024). More is different: mobile ions improve the design tolerances
of perovskite solar cells. Energy Environ. Sci..

[8] Torre
Cachafeiro M. A., Tress W. (2025). Ionic Losses and Gains in Perovskite
Solar Cells: Impact on Efficiency and Stability. ACS Energy Letters.

[9] Tress W., Correa Baena J. P., Saliba M., Abate A., Graetzel M. (2016). Inverted current–voltage
hysteresis in mixed perovskite solar cells: polarization, energy barriers,
and defect recombination. Adv. Energy Mater..

[10] Nemnes G. A., Besleaga C., Stancu V., Dogaru D. E., Leonat L. N., Pintilie L., Torfason K., Ilkov M., Manolescu A., Pintilie I. (2017). Normal and inverted
hysteresis in perovskite solar
cells. J. Phys. Chem. C.

[11] Wu F., Pathak R., Chen K., Wang G., Bahrami B., Zhang W.-H., Qiao Q. (2018). Inverted current–voltage
hysteresis
in perovskite solar cells. ACS Energy Letters.

[12] Clarke W., Cowley M. V., Wolf M. J., Cameron P., Walker A., Richardson G. (2023). Inverted hysteresis
as a diagnostic tool for perovskite
solar cells: Insights from the drift-diffusion model. J. Appl. Phys..

[13] Zhu P., Wang D., Zhang Y., Liang Z., Li J., Zeng J., Zhang J., Xu Y., Wu S., Liu Z. (2024). Aqueous synthesis of
perovskite precursors for highly
efficient perovskite solar cells. Science.

[14] Dong B., Wei M., Li Y., Yang Y., Ma W., Zhang Y., Ran Y., Cui M., Su Z., Fan Q. (2025). Self-assembled
bilayer for perovskite solar cells with improved tolerance against
thermal stresses. Nature Energy.

[15] Calado P., Telford A. M., Bryant D., Li X., Nelson J., O’Regan B. C., Barnes P. R. (2016). Evidence for ion
migration in hybrid
perovskite solar cells with minimal hysteresis. Nat. Commun..

[16] Thiesbrummel J., Shah S., Gutierrez-Partida E., Zu F., Peña-Camargo F., Zeiske S., Diekmann J., Ye F., Peters K. P., Brinkmann K. O. (2024). Ion-induced field screening
as a dominant factor
in perovskite solar cell operational stability. Nature Energy.

[17] Shen H., Jacobs D. A., Wu Y., Duong T., Peng J., Wen X., Fu X., Karuturi S. K., White T. P., Weber K. (2017). Inverted hysteresis in CH3NH3PbI3 solar cells: role of stoichiometry
and band alignment. J. Phys. Chem. Lett..

[18] Jacobs D. A., Wu Y., Shen H., Barugkin C., Beck F. J., White T. P., Weber K., Catchpole K. R. (2017). Hysteresis phenomena in perovskite
solar cells: the many and varied effects of ionic accumulation. Phys. Chem. Chem. Phys..

[19] Almora O., Lopez-Varo P., Cho K. T., Aghazada S., Meng W., Hou Y., Echeverría-Arrondo C., Zimmermann I., Matt G. J., Jiménez-Tejada J. A. (2019). Ionic
dipolar switching hinders charge collection in perovskite solar cells
with normal and inverted hysteresis. Sol. Energy
Mater. Sol. Cells.

[20] Alvarez A. O., Arcas R., Aranda C. A., Bethencourt L., Mas-Marzá E., Saliba M., Fabregat-Santiago F. (2020). Negative capacitance
and inverted hysteresis: matching features in perovskite solar cells. journal of physical chemistry letters.

[21] Torre
Cachafeiro M. A., Comi E. L., Parayil Shaji S., Narbey S., Jenatsch S., Knapp E., Tress W. (2025). Ion migration
in mesoscopic perovskite solar cells: Effects on electroluminescence,
open circuit voltage, and photovoltaic quantum efficiency. Adv. Energy Mater..

[22] Bertoluzzi L., Boyd C. C., Rolston N., Xu J., Prasanna R., O’Regan B. C., McGehee M. D. (2020). Mobile ion concentration measurement
and open-access band diagram simulation platform for halide perovskite
solar cells. Joule.

[23] Betancur P. F., Sohmer M., Mora-Seró I., Etgar L., Boix P. P. (2025). Working
Mechanisms of Triple-Oxide Mesoporous Hole-Transport-Layer-Free Printable
Perovskite Solar Cells via Impedance Spectroscopy. J. Phys. Chem. Lett..

[24] Rong Y., Hu Y., Ravishankar S., Liu H., Hou X., Sheng Y., Mei A., Wang Q., Li D., Xu M. (2017). Tunable
hysteresis effect for perovskite solar cells. Energy Environ. Sci..

[25] Luo X., Liu X., Lin X., Wu T., Wang Y., Han Q., Wu Y., Segawa H., Han L. (2024). Recent advances of inverted perovskite
solar cells. ACS Energy Letters.

