# Supplementary material for: Inverted J–V Hysteresis in Perovskite Solar Cells: Insights from Photovoltaic Quantum Efficiency
Source: ACS Energy Lett. 2026 Jan 30;11(2):2173–8. doi: 10.1021/acsenergylett.5c04035 (PMC12910652; doi:10.1021/acsenergylett.5c04035)
Supplement: Supplementary file 1 [file nz5c04035_si_001.pdf]

Supporting Information (SI): Inverted J–V Hysteresis in Perovskite Solar Cells: Insights from Photovoltaic Quantum Efficiency

Miguel Torre Cachafeiro[1,2], Carys A. Worsley[3], Fuxiang Ji[1], Trystan M. Watson[3], and Wolfgang Tress*[1]

[1]*Institute of Computational Physics, Zurich University of Applied Sciences (ZHAW), 8400 Winterthur, Switzerland*
[2]*Institut des Matériaux, École Polytechnique Fédérale de Lausanne (EPFL), 1015 Lausanne, Switzerland*
[3]*Swansea University, Bay Campus, Neath, Skewen SA18EN, Wales*

Contents

S1 General simulations **2**
S1.1 Supplementary Note 1: Effect of recombination mechanism 2

S2 HTL-free triple mesoscopic C-PSC **5**
S2.1 Supplementary Note 2: Effect of preconditioning time and role of m-TiO_2 layer 5

S3 Planar P-I-N PSC **13**

S4 Additional N-I-P PSCs with normal hysteresis **15**

S5 Reverse bias preconditioning **17**
S5.1 Supplementary Note 3: inverted hysteresis after reverse bias in C-PSCs 17

S6 Hysteresis direction diagnostic **19**

S7 Fabrication details **19**

*Email: wolfgang.tress@zhaw.ch

S1 General simulations

S1.1 Supplementary Note 1: Effect of recombination mechanism

Throughout this work, the effect of different ionic distributions is analyzed by using preconditioning voltages. This is illustrated by the simulations in Fig. S1, which are only meant to represent a general example to discuss the methodology used. The parameters used for this simulation are summarized in Table S1. Fig. S1a shows the steady state ionic distribution for different precondition voltages. For this parameter set, the 'ion-free' state - where ionic charge is mostly compensated in the bulk - occurs at a relatively low voltage (0.55 V), below V_{OC}. As a result, a 1.1 V precondition leads to substantial ionic accumulation of opposite polarity relative to the 0 V-conditioned case. It is important to note that in such cases, a fast *J*-*V* scan following a V_{OC} precondition does not reflect the 'ion-free' device performance[1]. As we have recently discussed in ref. [1], accumulated ions around the V_{OC} precondition can lead to either higher or lower performance compared to the equivalent 'ion-free' device, depending on device-specific properties[2;3]. In experiment, the steady state situation may take significant time to be reached due to the slow formation of ionic space charge layers[4;5]. This will introduce a time-dependence to the effect of preconditioning. Furthermore, the changes in ionic space charge layers may occur due to accumulation and depletion of cations (iodine vacancies) in the one mobile ion model, the generation of mobile ions[6] or the additional accumulation of slower cations and anions (e.g. methylammonium vacancies or iodine interstitials), which may be mobile at different timescales and introduce additional preconditioning-time dependencies, as we recently explored for C-PSCs[7].

Fig. S1b compares different scenarios of how J_{SC} is affected by the ionic charge distribution (precondition). The different scenarios are defined by the dominant recombination mechanism: scenario 'A' is dominated by bulk Shockley–Read–Hall (SRH) recombination, 'B' considers both bulk and interface SRH, and 'C' only interface SRH. High rates of interface recombination are considered to exaggerate the trends. In case 'A', higher precondition voltages always lead to higher J_{SC} (for the plotted range), whereas in case 'B' the maximum J_{SC} is reached for an intermediate precondition voltage. Finally, case 'C' shows a J_{SC} maximum closer to the 0 V precondition, with a significantly higher J_{SC} loss for the 1.1 V than the 0 V precondition. In this example, the J_{SC} loss with higher positive preconditions originates from enhanced interface recombination, whereas the loss under lower positive preconditions results from increased bulk SRH recombination. Negative preconditioning voltages lead to excessive ionic screening in the bulk (bulk field reversal), causing an abrupt decrease in J_{SC} across all cases. Upon a sudden change from applied 1.1 V to 0 V, the ionic charge will go through the distributions in Fig. S1a within the 1.1 V to 0 V precondition range. Since the ionic response is slow, its effect on current collection can be seen in the J_{SC} transient (Fig. S1c), which as expected shows the same trend as the precondition-dependent J_{SC} (Fig. S1b). In case 'A', J_{SC} decreases over time due to ionic relaxation, while in case 'C' it mostly increases. Case 'B', which is a combination of 'A' and 'C', initially shows a rising J_{SC}, due to reducing interface recombination as in case 'C', followed by the same decline of 'A' due to enhanced bulk SRH with ionic redistribution. Thus, from the transients in Fig. S1c it is natural to expect that when carrying out a voltage scan from 1.1 V in the BW direction, cases 'B' and 'C' will show higher current later in time (during the FW scan), whereas the opposite should happen in case 'A'. This is illustrated in Fig. S1d-e for a 10 mV s^{-1} scan. At low applied voltages in the *J*-*V* curve, cases 'B' and 'C' show inverted hysteresis (Fig. S1f), while case 'A' shows normal hysteresis. At higher voltages, the effect of the ionic distribution on V_{OC} can dominate. The dependence of the V_{OC} on the ionic distribution may oppose that of the J_{SC}, wich can lead to a crossover in the *J*-*V* curve, as seen in cases 'B' and 'C' where the drop in V_{OC} during the FW scan causes the hysteresis to switch from inverted to normal. In this study, *J*-*V* hysteresis directions are analyzed based on how current collection is influenced by the ionic distribution, which depends on the device properties and dominant recombination mechanisms, investigated via spectrally resolved EQE measurements[8]. In the main text, we present experimental results which can be related to the scenarios illustrated in Fig. S1. The results for the C-PSC without a mesoporous ETL correspond to case 'A', while the standard C-PSC aligns closely with case 'B'. Lastly, the planar p-i-n sample matches case 'C'.

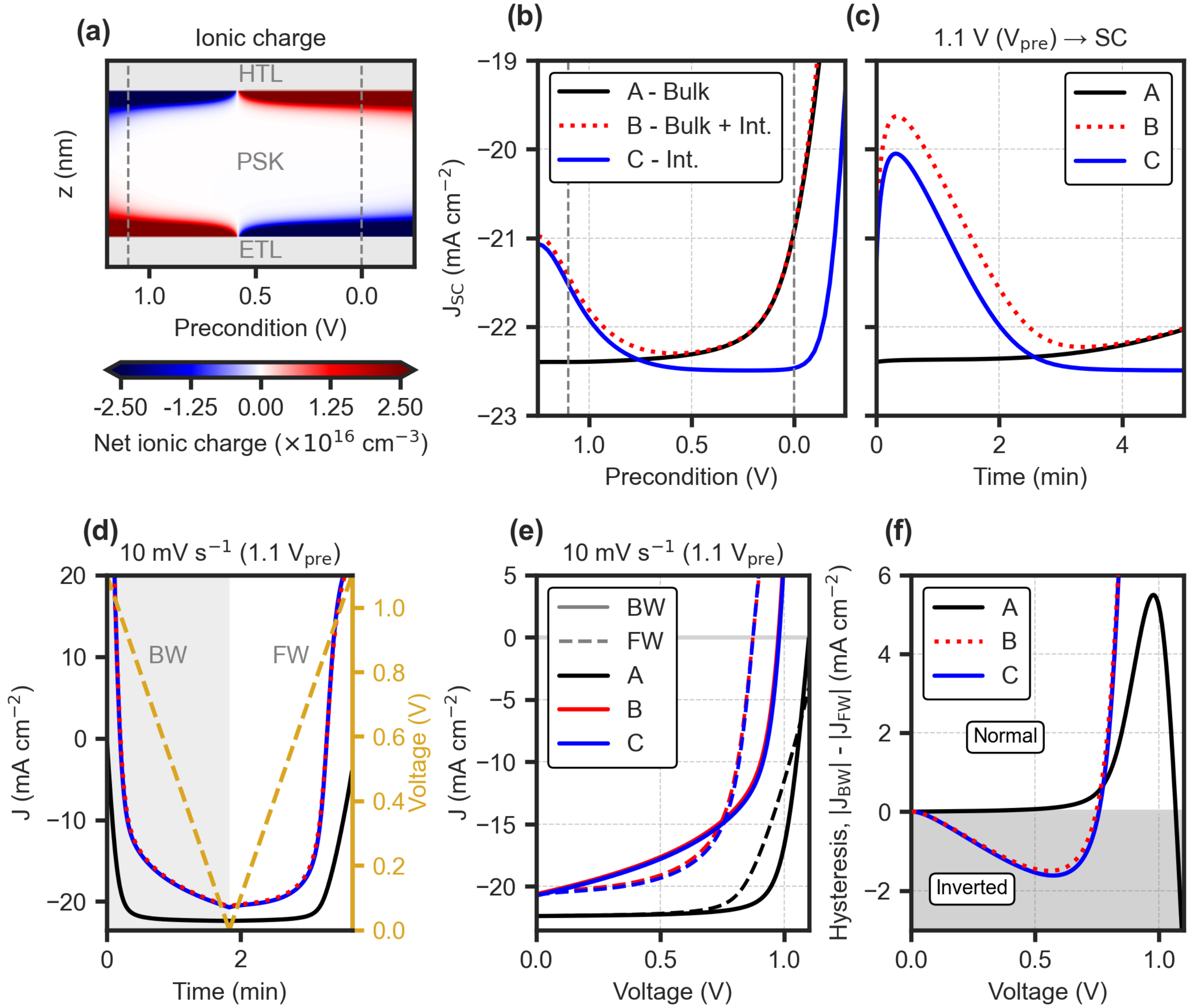

Figure S1: Simplified PSC device simulations including mobile ions. Different scenarios 'A', 'B' and 'C' correspond to different device properties, determined by different dominant recombination mechanisms. (a) Net ionic charge distribution for different precondition voltages (steady state). (b) Dependence of J_{SC} on precondition voltage. (c) Transient J_{SC} for slow ion mobility (1×10^{-11} cm^2 V^{-1} s^{-1}), after an abrupt change from 1.1 V to 0 V (SC). (d) Voltage scan from 1.1 V to 0 V (BW) and back up to 1.1 V (FW), at 10 mV s^{-1}. (e) Resulting J-V curve and (f) hysteresis computed until one of the scans first reaches V_{OC}.

Table S1: Parameters used in the introductory 1D PSC simulation. The model represents a generic PSC with mobile ions confined to the perovskite layer, which is sandwiched between charge selective layers. To compute the generation profile under AM1.5G illumination, complex refractive index (n-k) data from the Setfos database was used, for common PSC materials (FTO, TiO_2, $MAPbI_3$, Spiro-MeOTAD and Au.)

Parameter	**TOP**	**ETL**	**PSK**	**HTL**	**BOT**
Thickness, d [nm]	50	10	500	10	50
Work function, WF [eV]	4.5				5.1
Valence band energy, VB [eV]		7.5	5.6	5.3	
Conduction band energy, CB [eV]		4.3	4.0	2.1	
Density of states, DOS_{VB} [cm^{-3}]		10^{21}	10^{19}	10^{21}	
Density of states, DOS_{CB} [cm^{-3}]		10^{21}	10^{19}	10^{21}	
Electron mobility, μ_n [cm^{2}V^{-1}s^{-1}]		10^{-3}	1		
Hole mobility, μ_p [cm^{2}V^{-1}s^{-1}]			1	10^{-3}	
Anion mobility, μ_a [cm^{2}V^{-1}s^{-1}]			$5 \cdot 10^{-10}$		
Cation mobility, μ_c [cm^{2}V^{-1}s^{-1}]			$5 \cdot 10^{-10}$		
Dielectric constant, ε		25	25	25	
Anion density, N_a [cm^{-3}]			10^{17}		
Cation density, N_c [cm^{-3}]			10^{17}		
Bimolecular rec. prefactor, β_γ [cm$^3 s^{-1}$]			10^{-10}		
SRH lifetimes, τ_n and τ_p [ns]			100 (A&B)		
Interface rec. velocity, $v_{int,SRH}$ [cm s^{-1}]			100 (A&C)		

S2 HTL-free triple mesoscopic C-PSC

S2.1 Supplementary Note 2: Effect of preconditioning time and role of m-TiO_2 layer

Triple mesoscopic C-PSCs are a promising solution for highly scalable and stable PSCs[9–14], avoiding the use of organic hole transport layers (HTL) with poor stability[15;16] or precious metal electrodes[17]. Fig. S2 shows scan rate-dependent J-V curves for a standard HTL-free C-PSC device comprised of FTO / TiO_2 / m-TiO_2-AVA-MAPI / m-ZrO_2-AVA-MAPI / Carbon. The J-V curves in Fig. S2a are measured from a 10 s V_{OC} precondition each time, starting with the BW scan. Fig. S2b shows the rate-dependent J-V response for the same cell after ≈ 30 min at V_{OC} under illumination. After the longer V_{OC} preconditioning, the J_{SC} difference between fast and slow scan rates increases, with fast scans showing lower J_{SC}, in contrast to what would be expected from ionic screening effects in a device with normal hysteresis[1;18]. As seen in Fig. S2c, showing the difference between the BW and FW curves extracted from Fig. S2a, the negatively signed hysteresis is initially considerably low (predominant normal hysteresis). After the longer V_{OC} preconditioning (Fig. S2b), inverted hysteresis becomes significantly more pronounced, as seen in Fig. S2d. The faster scan rates show complete inverted hysteresis, with a transition to normal hysteresis (J-V crossover) occurring at lower voltages with lower scan rates. These trends indicate that here, in contrast to the more common case, the V_{OC} precondition can negatively impact current collection.

To better understand the role of preconditioning, V_{OC} transients upon turning on the illumination are shown in Fig. S3a for a timespan of 30 min, for a standard device and a device without m-TiO_2 layer, which is introduced to elucidate the role of the mesoporous ETL. Fast J-V curves at 10 V s^{-1} periodically taken during the V_{OC} transient, which interrupt the transient measurements (but do not disturb the general trend in Fig. S3a) and allow to probe the state of the cell over time, are shown in Fig. S3b-c. Measuring fast before ions can redistribute mimics the effect of using a precondition voltage in simulations (where ions are kept fixed with a distribution calculated for a given applied voltage). Despite the initial changes in V_{OC} and fill factor, the J_{SC} of the m-TiO_2-free device does not show significant changes with preconditioning time. In contrast, for the standard device with m-TiO_2, the J_{SC} continuously decreases. Since the J-Vs are measured fast (10 V s^{-1}) before ions can respond, the increasing J_{SC} loss cannot be attributed to bulk ionic screening. Instead, it could be interpreted as a growing non-ionic loss[18–20]; however, this can be ruled out here, as the loss is fully reversible and occurs on the timescales of ionic redistribution. If most of the ionic charge is compensated in the bulk at V_{OC}, ionic screening can be avoided by measuring fast from V_{OC}[1;21]. In devices with normal hysteresis, the V_{OC}-conditioned ionic distribution remains beneficial for current collection[18] - as for the m-TiO_2-free device. However, in this case the observed J_{SC} loss in the standard device is aggravating over preconditioning time at V_{OC}, potentially due to the slow accumulation of ionic charge.

Upon switching from open-circuit to short-circuit, mobile ions drift back as a response to the change in electric field, where their screening effect on the current collection can be seen in the J_{SC} transients in Fig. S3d. For the device without m-TiO_2, the J_{SC} drops monotonically, indicating that ionic screening upon ion redistribution from the V_{OC} precondition leads to a reduction in the driving force for charge extraction, causing a dramatic J_{SC} decrease of ≈ 50%. This implies that the diffusion length is not high enough to compensate for the reduction in electric field, requiring a shorter path for efficient electron injection into the ETL, which is facilitated by the mesoporous ETL in the standard configuration[22]. Differences in infiltration quality may further exacerbate this trend, possibly resulting in poorer perovskite quality in the m-TiO_2-free device[23]. For the standard C-PSC, after switching from open-circuit to short-circuit, the (absolute) J_{SC} transient shows instead an initial increasing regime, up to a maximum from where it decreases (Fig. S3d). This is in agreement with the trend in Fig. S2b and S2d, where the fastest scans show inverted, but the slowest one reverts to normal hysteresis, corresponding to the slowest J-V curve timescales operating on the decreasing regime of the J_{SC} transient. Similarly, this means that for the faster J-V curves with inverted hysteresis, the devices are operating on the timescales of the increasing J_{SC} regime, as discussed in the main text. The experimental results for the standard C-PSC align with case 'B' in the introductory example (Fig. S1), whereas the m-TiO_2-free device resembles case 'A'.

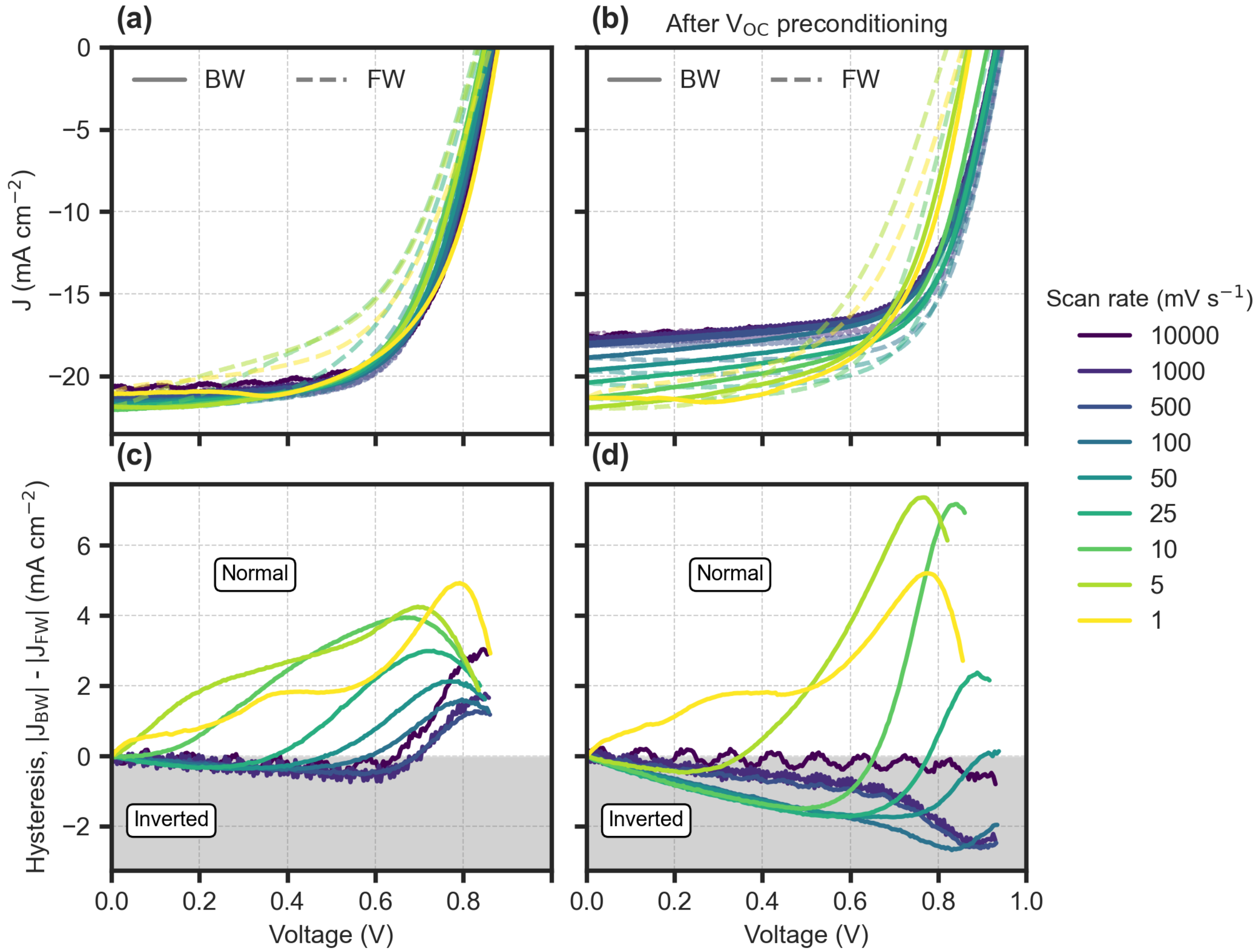

Figure S2: Impact of preconditioning at V_{OC}. J-V curves starting with the BW scan for varying scan rates, measured under AM1.5G solar simulator, for (a) initial state and (b) state after ≈ 30 minutes at V_{OC} under illumination. (c) Initial hysteresis and (d) after V_{OC} preconditioning, for the J-V curves above, where negative values correspond to higher current in the FW than in the BW scan.

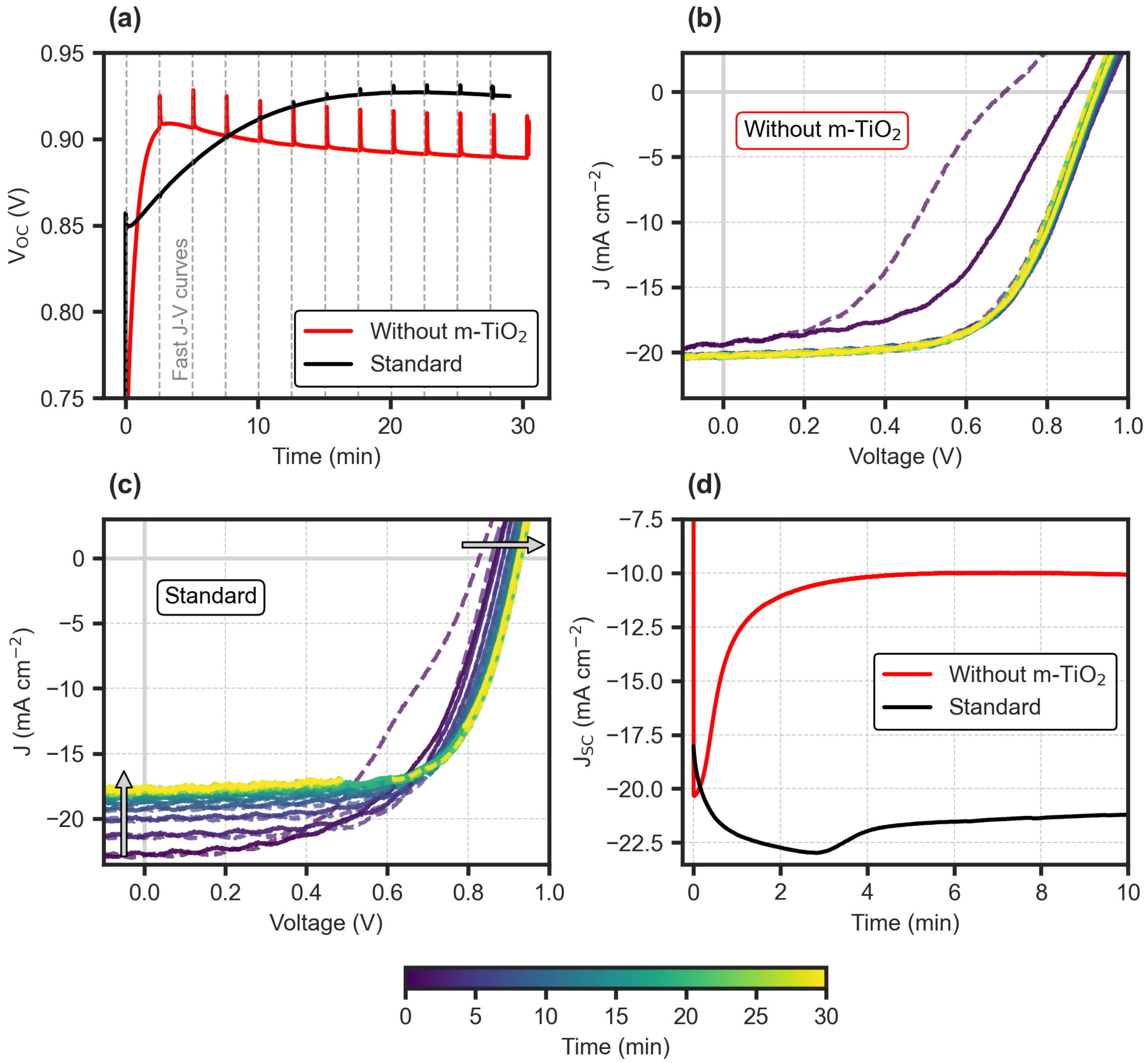

Figure S3: (a) Transient V_{OC} under AM1.5G solar simulator illumination, for a standard C-PSC and a device without m-TiO_2 layer. (b)-(c) Fast J-V scans (10 V s^{-1}) during the transients in (a). (d) J_{SC} transients upon switching from open-circuit to short-circuit under illumination, immediately following the transients in (a).

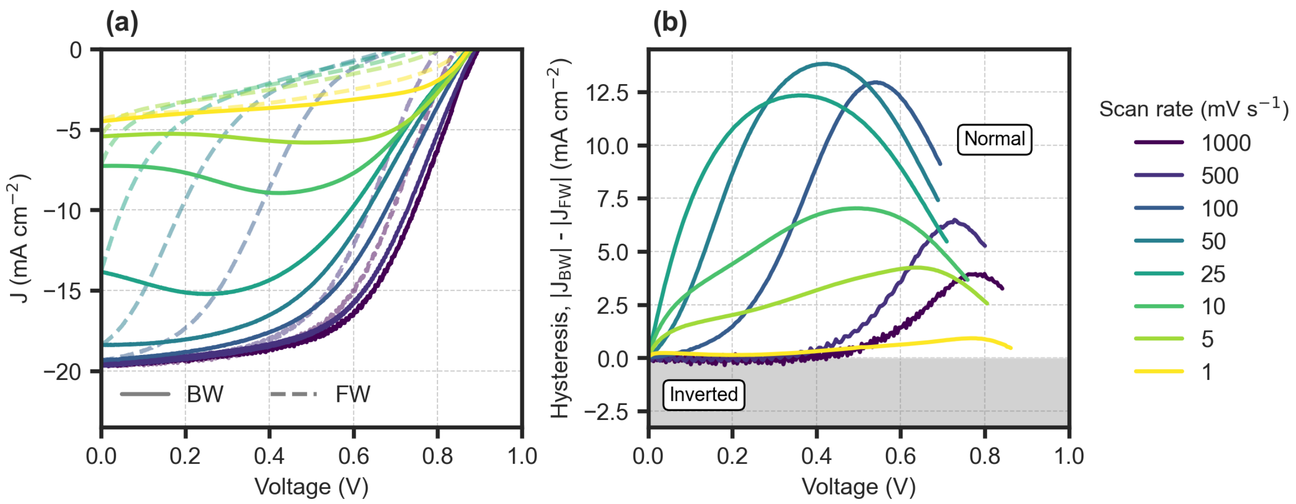

Figure S4: C-PSC device without m-TiO_2 layer. (a) *J-V* curves starting with the BW scan for varying scan rates, measured under AM1.5G solar simulator from a precondition at V_{OC} under illumination. (b) Hysteresis curves, where negative values correspond to higher current in the FW than in the BW scan. The m-TiO_2-free device shows no inverted hysteresis.

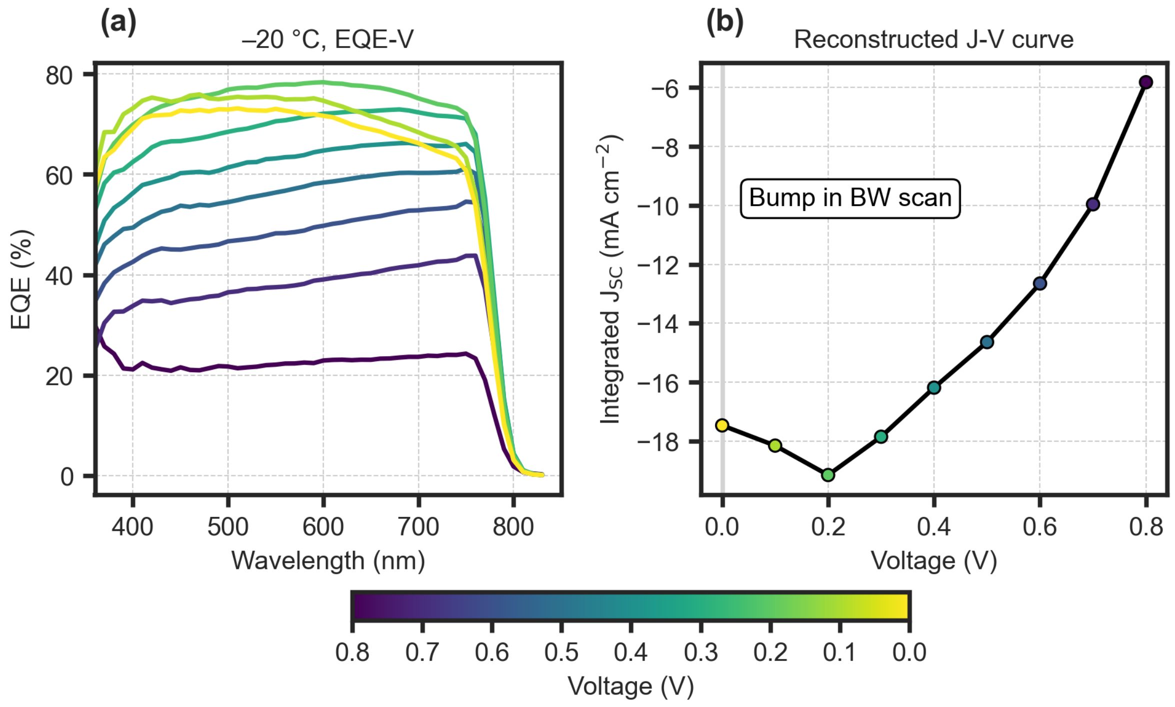

Figure S5: (a) Voltage-dependent EQE measured at low temperature (-20°C), cooling down at 1 V and starting with the BW scan voltages. Only the BW scan is shown. In this case, the temperature is high enough (mimicking a faster scan rate than in Fig. 4c-d in the main text) that ions can respond during the BW scan, all the way from the blue to the red loss. The drop in EQE in the red before reaching short-circuit, due to bulk ionic screening, results in the characteristic bump in the *J-V* curve of C-PSCs, as seen in (b) showing the integrated J(V) from the EQE(V) spectra in (a).

Table S2: Parameters used in the 1D C-PSC Setfos model. To compute the generation profile under AM1.5G illumination, complex refractive index (n-k) data from the Setfos database was used, for the corresponding materials (FTO, TiO_2, ZrO_2, $MAPbI_3$ and Carbon). For the mixed oxide-perovskite layers, the n-k data was mixed via an effective-medium approximation, assuming 50% m-TiO_2 and 55% m-ZrO_2 with the remaining made up of perovskite (the ratios are based on calculations for the cells used). For this model the voltage at which the net ionic charge reaches a minimum is 0.958 V ('ion-free' voltage).

Parameter	**FTO**	**TiO_2**	**m-TiO_2-MAPI**	**m-ZrO_2-MAPI**	**C**
Thickness, d [nm]	450	50	700	2000	10000
Work function, WF [eV]	4.1				5.2
Valence band energy, VB [eV]		6.0	5.4	5.4	
Conduction band energy, CB [eV]		4.1	3.9	3.9	
Density of states, DOS_{VB} [cm^{-3}]		10^{19}	10^{19}	10^{19}	
Density of states, DOS_{CB} [cm^{-3}]		10^{19}	10^{19}	10^{19}	
Electron mobility, μ_n [$cm^2V^{-1}s^{-1}$]		10^{-3}	1	1	
Hole mobility, μ_p [$cm^2V^{-1}s^{-1}$]		10^{-3}	1	1	
Anion mobility, μ_a [$cm^2V^{-1}s^{-1}$]			Static		
Cation mobility, μ_c [$cm^2V^{-1}s^{-1}$]			10^{-9}		
Dielectric constant, ε		60	35	35	
Anion density, N_a [cm^{-3}]			10^{16}		
Cation density, N_c [cm^{-3}]			10^{16}		
Bimolecular rec. prefactor, β_γ [cm^3s^{-1}]			10^{-10}	10^{-10}	
SRH lifetimes, τ_n and τ_p [ns]			500	500	
Interface rec. velocity, $v_{int,SRH}$ [cms^{-1}]			500		

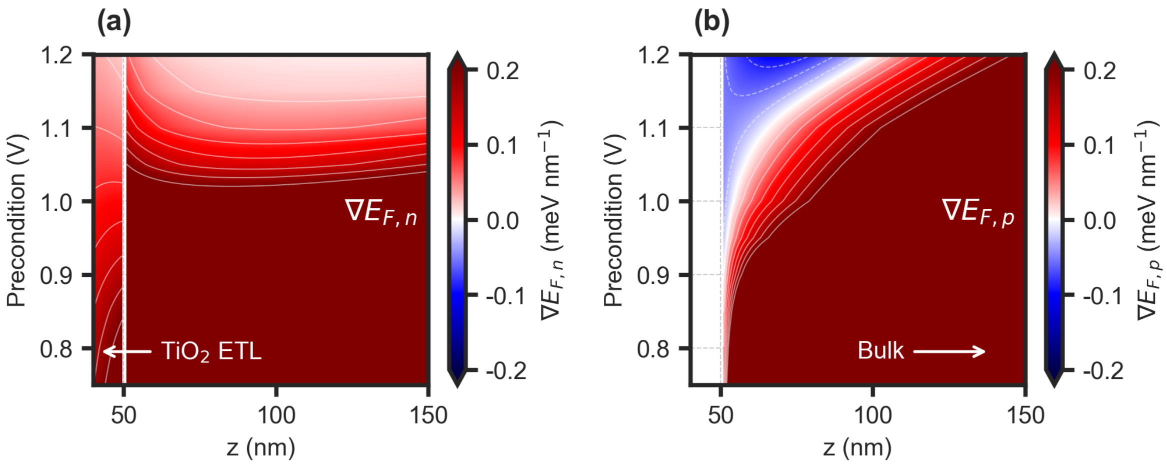

Figure S6: Simulated driving force as a function of depth (z), for (a) electrons and (b) holes at short-circuit, showing the region nearby the ETL-interface, focusing on the higher precondition voltages. The sign of ∇E_F is defined so that a positive gradient corresponds to a force pushing carriers toward their respective collecting electrodes, while a negative gradient drives them toward the wrong contact. Illumination is incident from the left (ETL) side.

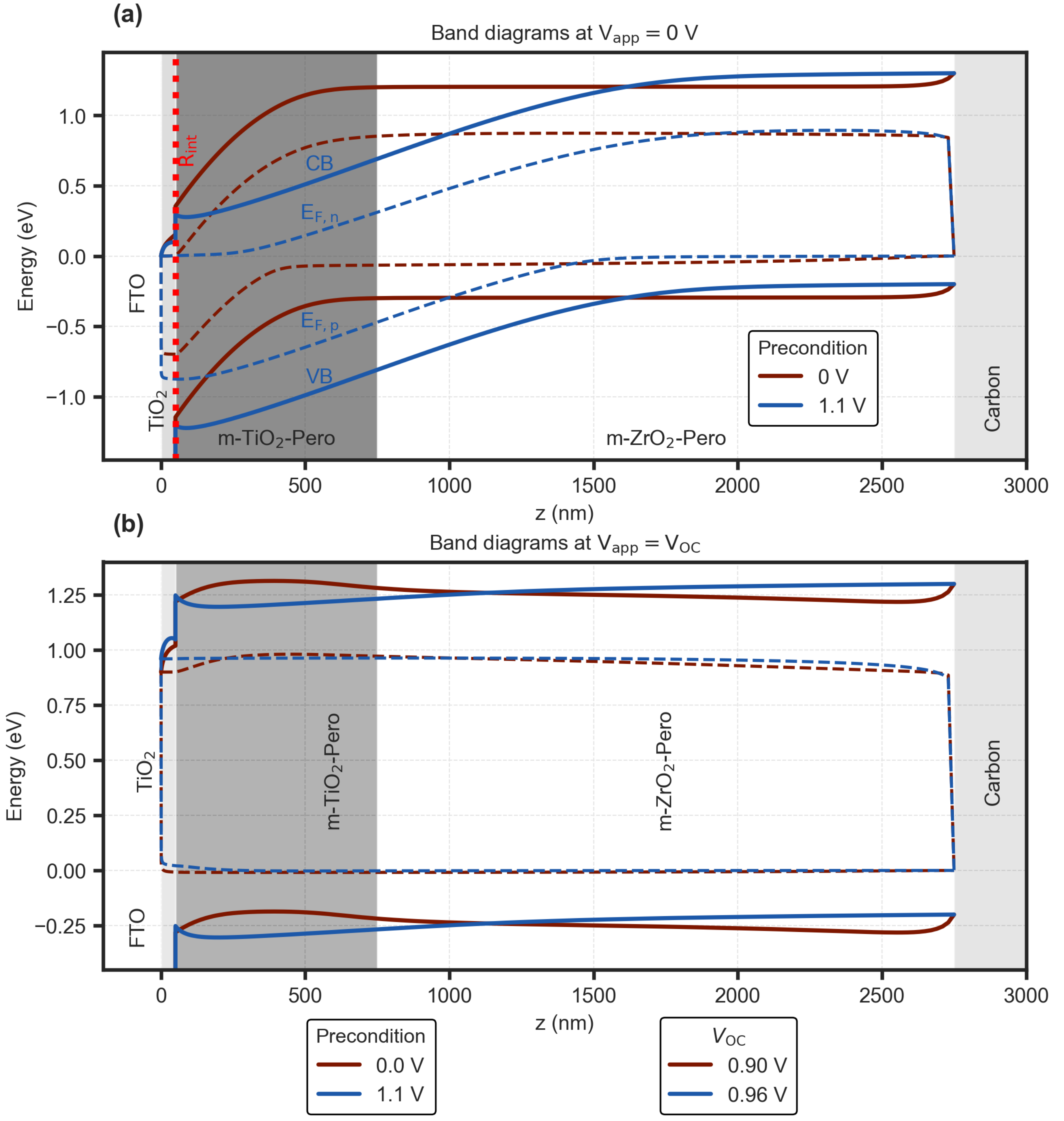

Figure S7: Simulated band diagrams along the device's depth (z) at (a) short-circuit and (b) open-circuit for the 0 V and 1.1 V preconditions.

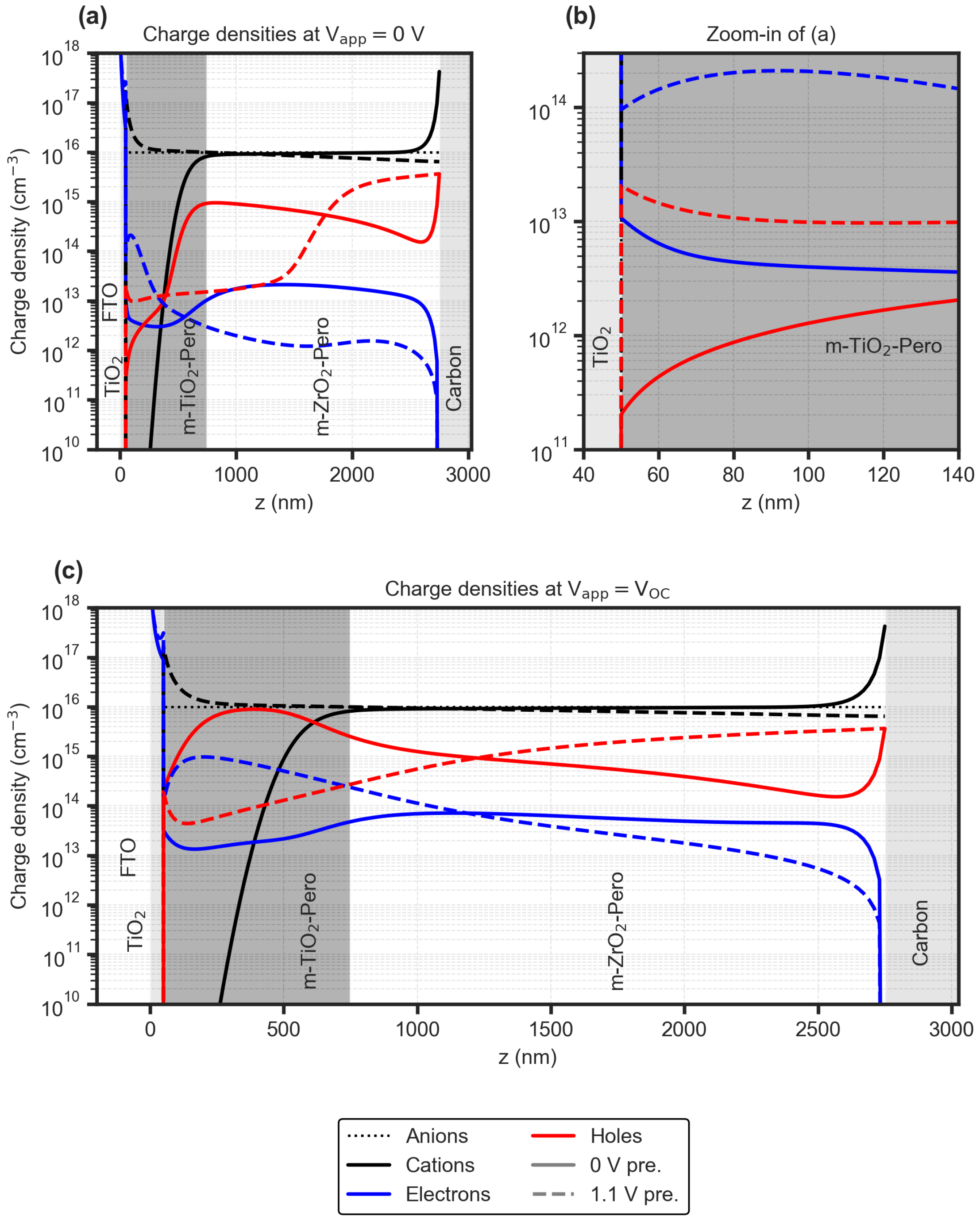

Figure S8: Simulated charge density profiles for a CPSC at (a)-(b) short circuit and (c) open circuit for different precondition voltages.

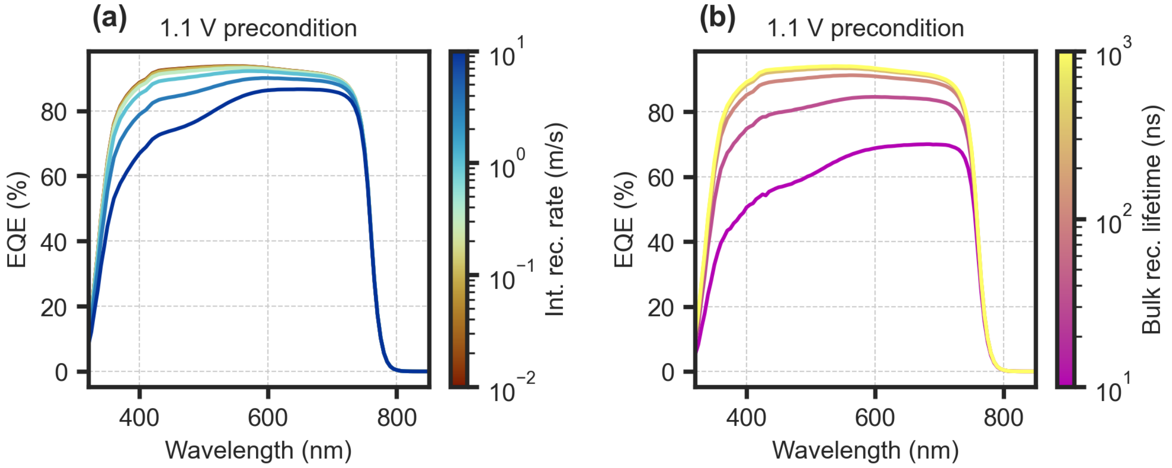

Figure S9: Simulated EQE spectra for the C-PSC with a 1.1 V precondition for ions. (a) EQE for a front-interface (ETL-perovskite) recombination velocity sweep, showing how the blue loss depends on the rate of recombination available at the front-side. (b) EQE for a bulk recombination rate sweep (SRH lifetimes), without any interface recombination. The EQE also tends to drop more in the blue with lowering the bulk SRH lifetime.

S3 Planar P-I-N PSC

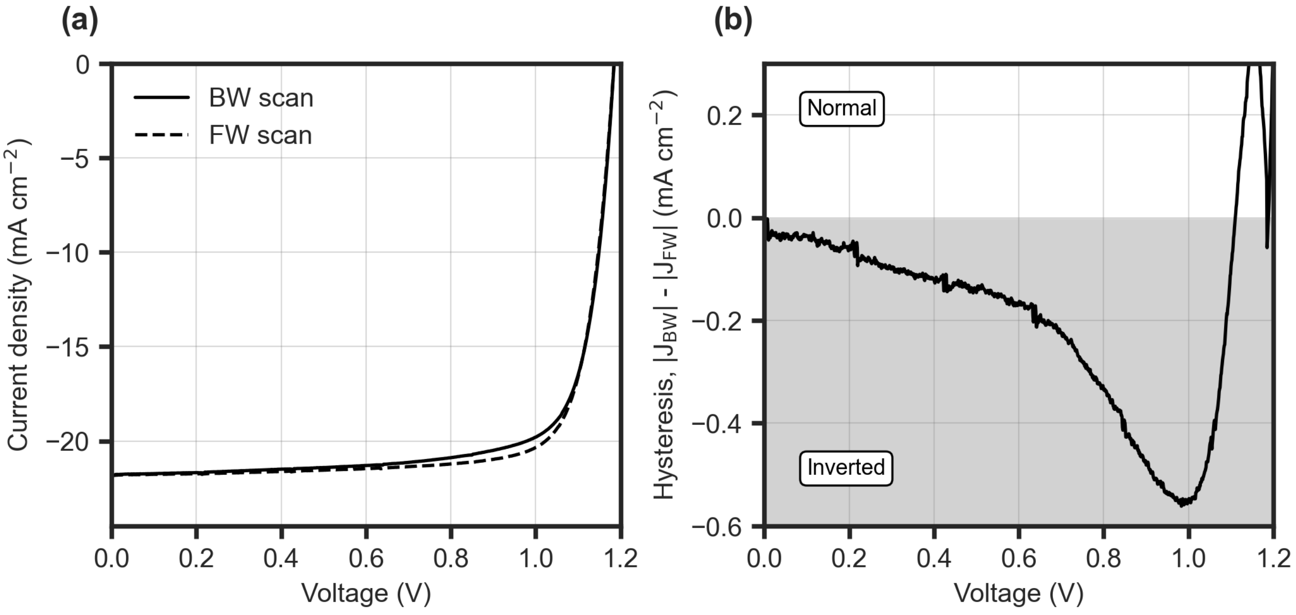

Figure S10: (a) J-V curve starting with the BW scan (100 mV s^{-1}) for a fresh p-i-n sample, measured under AM1.5G solar simulator, from a V_{OC} precondition. (b) Hysteresis curves from (a), showing dominant IH at this scan rate.

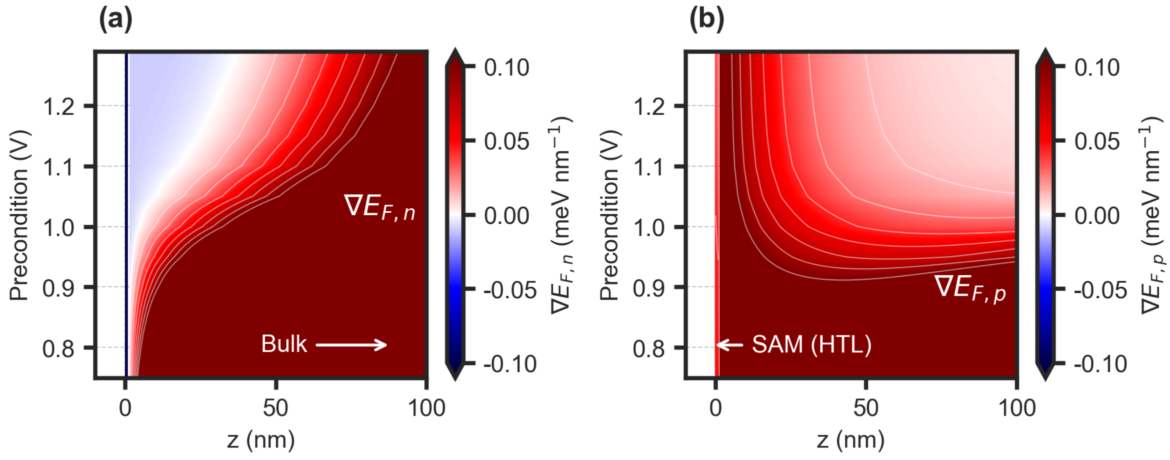

Figure S11: Simulated driving force as a function of depth (z), for (a) electrons and (b) holes at short-circuit, showing the region nearby the thin SAM HTL-interface, focusing on the higher precondition voltages. The sign of ∇E_F is defined so that a positive gradient corresponds to a force pushing carriers toward their respective collecting electrodes, while a negative gradient drives them toward the wrong contact. In this case, holes are extracted at the left (front) side, while electrons have to move toward the bulk to be reach the ETL at the back-electrode. Illumination is incident from the left (HTL) side.

Table S3: Parameters used in the 1D p-i-n PSC Setfos model. To compute the generation profile under AM1.5G illumination, complex refractive index (n-k) data from the Setfos database was used to approximate the experimental stack. (FTO, $MAPbI_3$, PCBM and Au). The SAM HTL was not considered in the optical model. For this model the voltage at which the net ionic charge reaches a minimum is 0.695 V ('ion-free' voltage).

Parameter	**FTO**	**HTL**	**PSK**	**ETL**	**Au**
Thickness, d [nm]	50	1	700	20	50
Work function, WF [eV]	5.2				4.5
Valence band energy, VB [eV]		5.35	5.5	7.5	
Conduction band energy, CB [eV]		2.0	4.0	4.3	
Density of states, DOS_{VB} [cm^{-3}]		10^{19}	10^{19}	10^{19}	
Density of states, DOS_{CB} [cm^{-3}]		10^{19}	10^{19}	10^{19}	
Electron mobility, μ_n [$cm^2V^{-1}s^{-1}$]			1	10^{-3}	
Hole mobility, μ_p [$cm^2V^{-1}s^{-1}$]		10^{-2}	1		
Anion mobility, μ_a [$cm^2V^{-1}s^{-1}$]			Static		
Cation mobility, μ_c [$cm^2V^{-1}s^{-1}$]			10^{-11}		
Dielectric constant, ε		5	15	5	
Anion density, N_a [cm^{-3}]			10^{16}		
Cation density, N_c [cm^{-3}]			10^{16}		
Bimolecular rec. prefactor, β_γ [cm^3s^{-1}]			10^{-10}		
SRH lifetimes, τ_n and τ_p [ns]			-		
Interface rec. velocity, $v_{int,SRH}$ [cms^{-1}]		100			

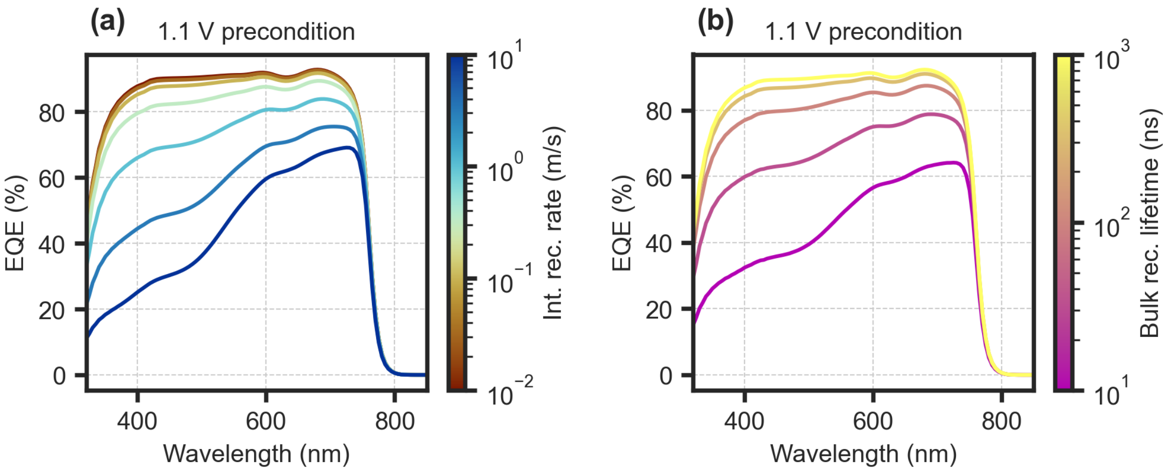

Figure S12: Simulated EQE spectra for the p-i-n cell with a 1.1 V precondition for ions. (a) EQE for a front-interface (HTL-perovskite) recombination velocity sweep, showing how the predominantly blue loss depends on the rate of recombination available at the front-side. (b) EQE for a bulk recombination rate sweep (bulk SRH lifetimes), without any interface recombination. The EQE also tends to drop more in the blue with lowering the bulk SRH lifetime.

S4 Additional N-I-P PSCs with normal hysteresis

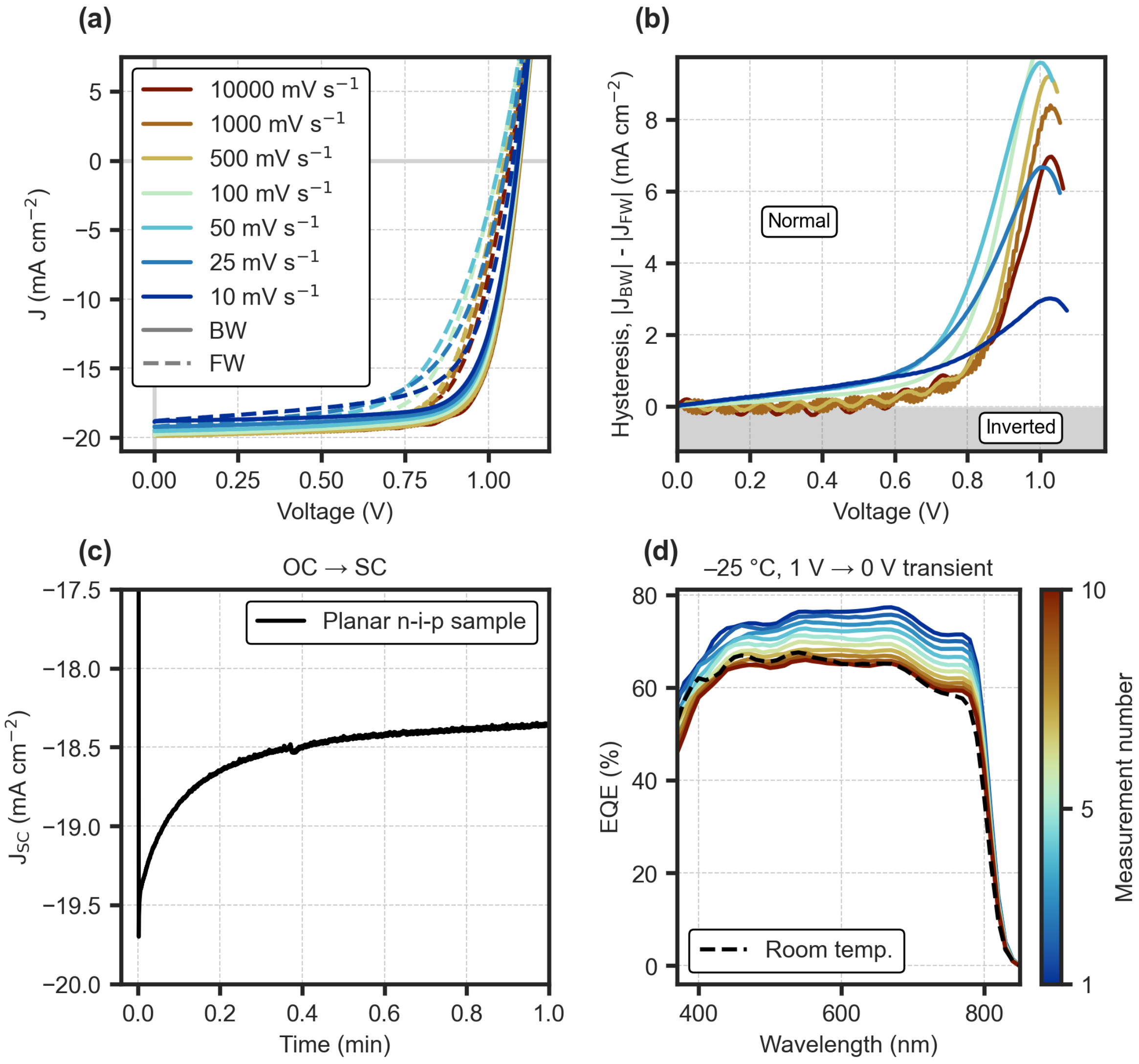

Figure S13: Experimental results for a fully planar n-i-p device (FTO / ALD-TiO_2 / SnO_2 / Perovskite / PEAI / Spiro-MeOTAD / Au) with normal hysteresis. The perovskite absorber composition is the same as for the p-i-n devices: $(FA_{0.95}MA_{0.05})_{0.95}\,Cs_{0.05}\,Pb\,(I_{0.95}Br_{0.05})_3$. (a) *J*-*V* curves starting with the BW scan for varying scan rates, measured under AM1.5G solar simulator, from a V_{OC} precondition. (b) Hysteresis curves from (a), computed until one of the scans first reaches V_{OC}, showing normal hysteresis only. (c) Transient J_{SC} upon switching from open-circuit (OC) to short-circuit (SC). (d) Sequential EQE measurements at short-circuit at low temperature (-25°C), from a precondition close to V_{OC} level (1 V), applied while cooling down.

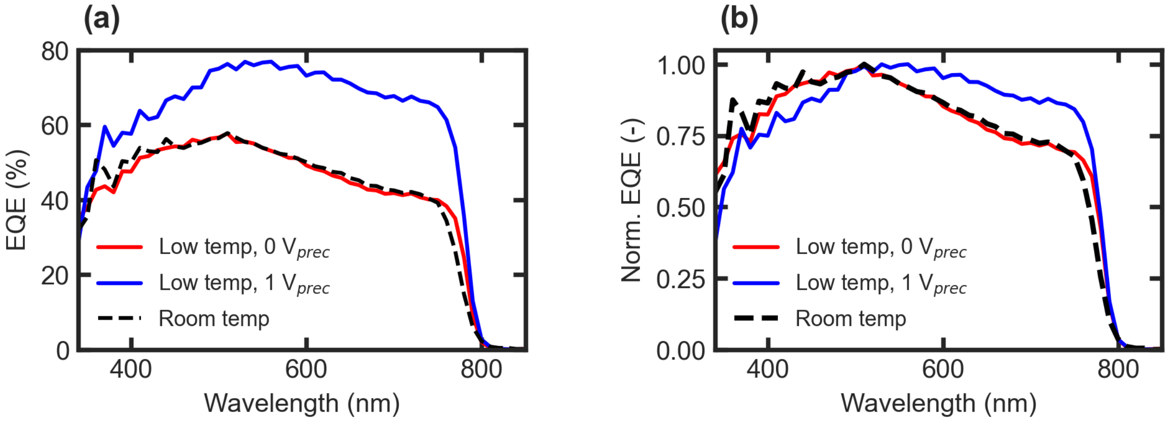

Figure S14: (a) Experimental EQE spectra for a semi-planar n-i-p architecture comprised of FTO / c-TiO_2 / m-TiO_2 / Perovskite / Spiro-MeOTAD / Au, showing high ionic current losses with normal hysteresis. Measured at short-circuit at low temperature (-50°C) while cooling down at 0 V or 1 V, and compared with the room temperature EQE. (b) Normalized EQE spectra, showing how the relative increase for the 1 V precondition is lower in the blue (higher in the red).

S5 Reverse bias preconditioning

S5.1 Supplementary Note 3: inverted hysteresis after reverse bias in C-PSCs

A high positive bias precondition can induce inverted hysteresis, due to the potentially detrimental impact of ionic accumulation on current collection, with characteristic blue losses in EQE. For a negative bias precondition ($V_{\mathrm{pre}} < 0$), for which inverted hysteresis can also be induced,[24] the J_{SC} at short-circuit will also tend to increase from the reverse bias precondition. This is illustrated in Fig. S15 showing the increasing trend in the short-circuit EQE after a prolonged reverse bias pre-stressing (-1.2 V, $\approx$ 16 h) for a C-PSC, initially showing a dramatic drop in the red due to the bulk field inversion, caused by the reverse bias precondition of ionic charge. This is accompanied by high collection probability for charges generated at the front interface, despite the reverse bias precondition, as seen by the mostly unaffected or even higher EQE in the blue. Under such conditions, the direction of hysteresis will again depend on the scan rate, on which scan direction is measured first, and on whether the recovery process from the detrimental reverse bias state or the instantaneous ionic response during the scan dominates. Interestingly, the transient in this case can be resolved at room temperature, due to the considerably slow recovery process after the prolonged application of the reverse bias voltage. However, the specific underlying reasons for the slower transients are beyond the scope of this work, and the reverse bias precondition is only briefly discussed here to address previous observations of inverted hysteresis from $V_{\mathrm{pre}} < 0$. The EQE spectra were measured after a reverse bias stability test, under which C-PSCs show promising stability.[25] However, shorter preconditioning times can already induce the relevant changes. Fig. S15b shows the J_{SC} transient for a C-PSC after just 1 minute under -1 V. J-V curves measured from the same precondition, starting with the BW scan, are shown in Fig. S15c. Intermediate scan rates show completely inverted hysteresis (Fig. S15c-d), due to the dominant effect of the recovery process (ionic relaxation enhancing current collection).

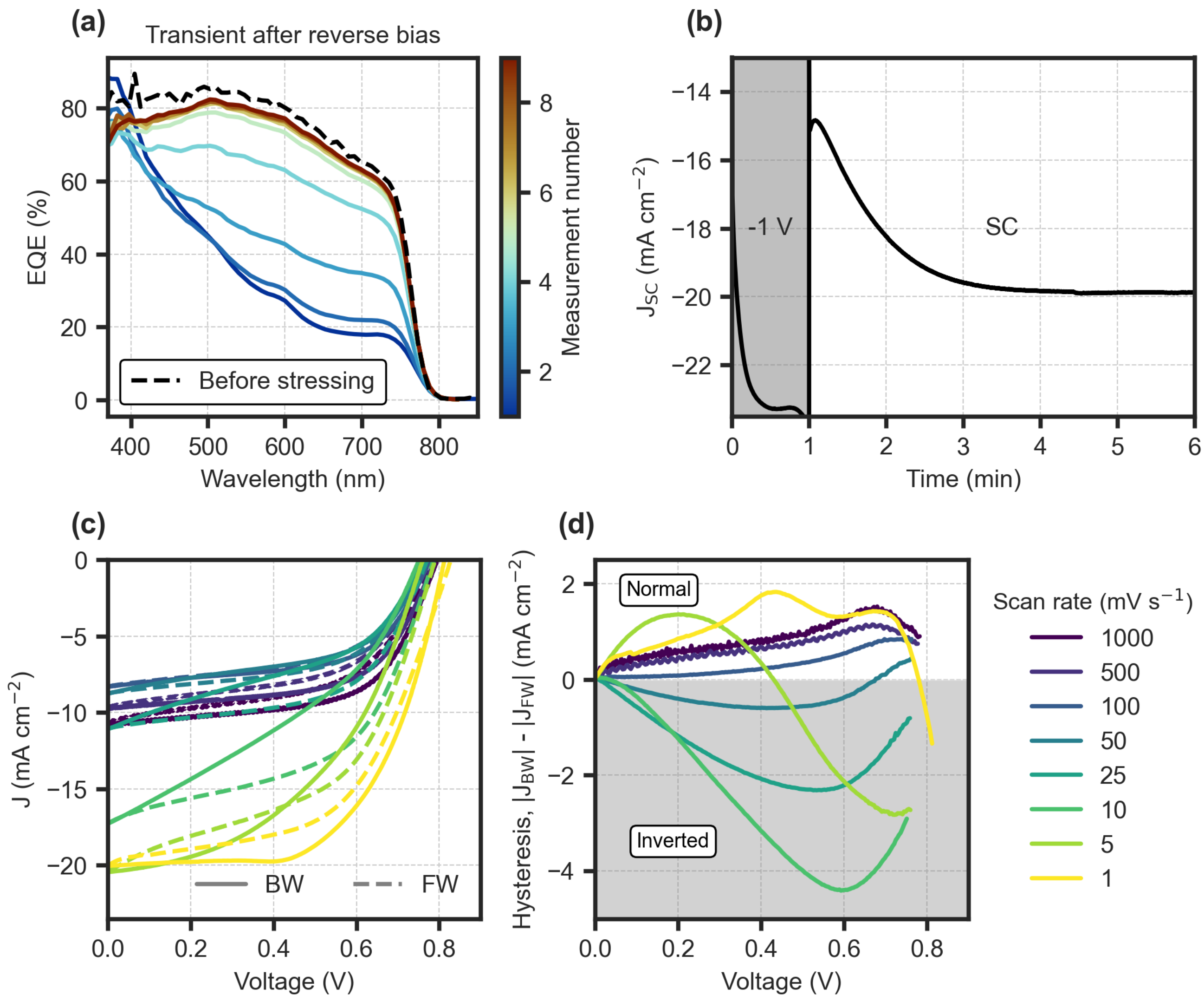

Figure S15: Reverse bias preconditioning of a C-PSC. (a) Sequential EQE measurements at short-circuit, measured at room temperature after ≈16 h-long prebiasing at -1.2 V, for a C-PSC. (b) J_{SC} transient under AM1.5G solar simulator illumination, for a switch from -1 V (1 min) to short-circuit. (c) Scan rate-dependent J-V curves measured after a 1 min precondition at -1 V each time, starting with the BW scan. (d) Hysteresis extracted from the J-V curves in (c).

S6 Hysteresis direction diagnostic

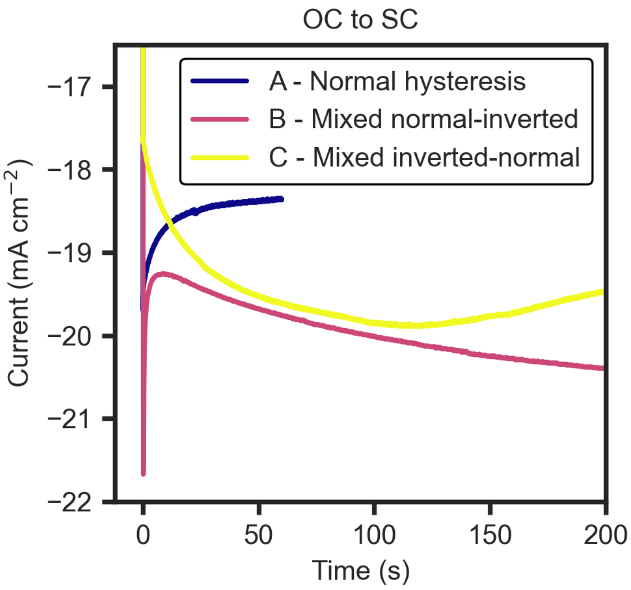

Figure S16: Experimental J_{SC} transients from a V_{OC} precondition, showing different trends. As discussed in the main text, the rate-dependent direction of the hysteresis in J-V curves measured from a V_{OC} precondition, starting with the BW scan, can be readily understood from the transients shown here. Type A, measured for a planar n-i-p device, represents a device with normal hysteresis only, since the J_{SC} transient decays monotonically with ionic screening. Type B, corresponding to the planar p-i-n device, leads to normal hysteresis at faster scan rates, followed by inverted one at slower scan rates. Type C, measured for the mesoscopic C-PSCs, results in inverted hysteresis followed by normal one at slower scan rates.

S7 Fabrication details

Triple mesoscopic C-PSC

Materials:

Substrates consisted of FTO Tc7 glass (XOP). Stack materials consisted of: Titanium diisopropoxide bis(acetylacetonate) (TAA, 75% in IPA, Sigma-Aldrich), anhydrous 2-propanol (IPA, 99.5%, Sigma-Aldrich), TiO_2 paste (30NR-D, GreatCell Solar), ZrO_2 paste (GreatCell Solar), carbon paste (Gwent Electronic Materials), and terpineol (95%, Sigma-Aldrich) were used as received. Precursor materials included PbI_2 (99%, TCI), MAI (CH_3NH_3I, anhydrous, Dyesol), 5-ammonium valeric acid iodide (5-AVAI, Dyesol), γ-valerolactone (GVL, Sigma-Aldrich), and anhydrous MeOH (99%, Sigma-Aldrich), and were used as received.

Device fabrication:

FTO substrates were patterned with a Nb/YVO_4 laser (532 nm) before cleaning with ~2% Hellmanex in deionized water, rinsing with acetone and IPA, and drying with N_2. Substrates were then placed in a Nano plasma system (Diener Electronics), and cleaned for 10 min in an O_2 environment. The substrate was heated to 300 °C on a hot plate, and a compact TiO_2 blocking layer (~50 nm) was deposited by spray pyrolysis of 0.2 M titanium di-isopropoxide-bis(acetylacetonate) in IPA. To form the mesoporous TiO_2 layer, the titania paste (30NRD) was diluted 1:1 by weight in terpineol, screen printed, and sintered at 550 °C for 30 min after a slow ramp. This step was omitted for the m-TiO_2-free devices. Next, ZrO_2 and carbon were printed and annealed at 400 °C for

30 min each. (1) Layer thicknesses were 600–800 nm, ∼2–2.2 μm, and ∼12–15 μm for TiO_2, ZrO_2, and carbon, respectively. All layers were printed and annealed in ambient conditions. The $AVA_{0.03}MAPbI_3$ precursors were prepared by dissolving 0.0086 g 5-AVAI, 0.1753 g MAI, and 0.5062 g PbI_2 in a mixture of 0.9 mL GVL and 0.1 mL MeOH. All precursors were fabricated in an N_2 glovebox and stirred at room temperature until dissolved. Once fabricated, precursors were stored in dark ambient conditions (∼30–50% RH, 18–21 °C). Devices were cooled to room temperature in ambient conditions (∼30–50% RH, 18–21 °C) before drop casting of 18 μL of room temperature precursor onto the stack surface. Devices were left for 22 min in ambient conditions after drop casting the precursor to ensure adequate infiltration before annealing on a hot plate for 1 h at 50 or 45 °C under a partial cover. Contacts were applied with an ultrasonic solder at 180 °C the day after infiltration. Encapsulated samples were prepared using butyl rubber tape around the device perimeter. Wires were sandwiched between two layers of tape, before placing a clean glass cover, and placing cells in a medium pressure T-shirt press at room temperature overnight to enable butyl rubber spread.

Planar P-I-N PSC

Materials:

Lead iodide (PbI_2) (99.99%), [4-(3,6-Dimethoxy-9H-carbazol-9-yl)butyl]phosphonic acid (MeO-4PACZ) (≥98.0%), Bathocuproine (BCP) (purified by sublimation) (>99.0%) and piperazine dihydriodide (PZ·2HI) (>98.0%) were purchased from TCI. Formamidinium iodide (FAI) (≥99%, anhydrous), methylammonium iodide (MAI) (≥99%, anhydrous), lead bromide ($PbBr_2$), methylammonium bromide (MABr) (≥99%, anhydrous), methylammonium chloride (MACl) (≥99%, anhydrous), cesium iodide (CsI) (99.999% trace metals basis, perovskite grade), dimethyl sulfoxide (DMSO) (anhydrous, ≥99.9%), N,N-dimethylformamide (DMF) (anhydrous, ≥99.8%), chlorobenzene (CB) (anhydrous, ≥99.8%), methanol (99.5%, anhydrous), isopropanol (IPA) (99.5%, anhydrous) were purchased from Sigma-Aldrich. Methyl [6,6]-phenyl-C_{61}-butyrate (PCBM) (99.9%) was purchased from Advanced Election Technology Co. Ltd.

Device fabrication:

The substrates were sequentially cleaned using soap, acetone, isopropanol, and ethanol to remove surface contaminants. The substrate was then cleaned with ultraviolet ozone for 30 min. Then, a solution of MeO-4PACZ was prepared by dissolving 0.5 mg of MeO-4PACZ in 1 mL of methanol, which was spin-coated on the substrate at 3000 rpm for 30 s and annealed at 100 °C for 10 min. The perovskite precursor solution with a molar concentration of 1.5 M was prepared by adding FAI, PbI_2, CsI, MABr and $PbBr_2$ into a mixed DMF/DMSO solvent with the ratio of 4:1 following the formula of $Cs_{0.05}(FA_{0.95}MA_{0.05})_{0.95}Pb(I_{0.95}Br_{0.05})_3$. An additional 5 mol% PbI_2 and 25 mol% MACl were added to the precursor. After stirring overnight, the precursor solution (45 μL) was spin coated at 2500 rpm for 10 s then at 5000 rpm for 40 s, with chlorobenzene dripped onto the film at 30 s before the end of the spin-coating. The film was finally annealed at 100 °C for 30 min. The passivation-layer solution was prepared by dissolving 1 mg of piperazine dihydriodide in 1 mL of isopropanol and stirring overnight. The solution was then dynamically spin-coated on the perovskite layer at 5000 rpm for 25 s and annealed at 100 °C for 10 min. After cooling, the film was washed with isopropanol under the same spin-coating conditions (5000 rpm, 25 s) and annealed again at 100 °C for 10 min. Then, the PCBM solution (20 mg/mL in chlorobenzene) was spin-coated on top of the interfacial passivation layer as an electron transport layer at 1500 rpm for 25 s and annealed at 70 °C for 10 min. Sequentially, the BCP solution in isopropanol was dynamically spin-coated on the PCBM layer at 5000 rpm for 30 s and annealed at 70 °C for 10 min. The device was completed by thermally evaporating the electrode of 60 nm Au.

References

[1] Torre Cachafeiro, M. A.; Tress, W. Ionic Losses and Gains in Perovskite Solar Cells: Impact on Efficiency and Stability. *ACS Energy Letters* **2025**, 4849–4855.

[2] Córdoba, M.; Taretto, K. Insight into the dependence of Photovoltaic Performance on Interfacial Energy Alignment in Solar cells with Mobile ions. *Solar RRL* **2024**, *8*, 2300742.

[3] Hart, L. J.; Angus, F. J.; Li, Y.; Khaleed, A.; Calado, P.; Durrant, J. R.; Djurišić, A. B.; Docampo, P.; Barnes, P. R. More is different: mobile ions improve the design tolerances of perovskite solar cells. *Energy & Environmental Science* **2024**, *17*, 7107–7118.

[4] Zhao, C.; Chen, B.; Qiao, X.; Luan, L.; Lu, K.; Hu, B. Revealing Underlying Processes Involved in Light Soaking Effects and Hysteresis Phenomena in Perovskite Solar Cells. *Advanced Energy Materials* **2015**, *5*.

[5] Pockett, A.; Raptis, D.; Meroni, S. M.; Baker, J.; Watson, T.; Carnie, M. Origin of exceptionally slow light soaking effect in mesoporous carbon perovskite solar cells with AVA additive. *The Journal of Physical Chemistry C* **2019**, *123*, 11414–11421.

[6] Xu, Z.; Kerner, R. A.; Kronik, L.; Rand, B. P. Beyond Ion Migration in Metal Halide Perovskites: Toward a Broader Photoelectrochemistry Perspective. *ACS Energy Letters* **2024**, *9*, 4645–4654.

[7] Torre Cachafeiro, M. A.; Comi, E. L.; Parayil Shaji, S.; Narbey, S.; Jenatsch, S.; Knapp, E.; Tress, W. Ion migration in mesoscopic perovskite solar cells: Effects on electroluminescence, open circuit voltage, and photovoltaic quantum efficiency. *Advanced Energy Materials* **2025**, *15*, 2403850.

[8] Abudulimu, A. et al. Bias-Dependent Quantum Efficiency Reveals Recombination Pathways in Thin Film Solar Cells. *Advanced Energy Materials* **2025**, *15*, e01709.

[9] Etgar, L.; Gao, P.; Xue, Z.; Peng, Q.; Chandiran, A. K.; Liu, B.; Nazeeruddin, M. K.; Gratzel, M. Mesoscopic CH3NH3PbI3/TiO2 heterojunction solar cells. *Journal of the American Chemical Society* **2012**, *134*, 17396–17399.

[10] Ku, Z.; Rong, Y.; Xu, M.; Liu, T.; Han, H. Full printable processed mesoscopic CH3NH3PbI3/TiO2 heterojunction solar cells with carbon counter electrode. *Scientific reports* **2013**, *3*, 3132.

[11] Mei, A.; Li, X.; Liu, L.; Ku, Z.; Liu, T.; Rong, Y.; Xu, M.; Hu, M.; Chen, J.; Yang, Y.; others A hole-conductor–free, fully printable mesoscopic perovskite solar cell with high stability. *Science* **2014**, *345*, 295–298.

[12] Grancini, G.; Roldán-Carmona, C.; Zimmermann, I.; Mosconi, E.; Lee, X.; Martineau, D.; Narbey, S.; Oswald, F.; De Angelis, F.; Graetzel, M.; others One-Year stable perovskite solar cells by 2D/3D interface engineering. *Nature communications* **2017**, *8*, 15684.

[13] Li, D.; Rong, Y.; Hu, Y.; Mei, A.; Han, H. Printable Mesoscopic Perovskite Solar Cells. 2021; http://dx.doi.org/10.1002/9783527825790.ch13.

[14] Liu, J.; Chen, X.; Chen, K.; Tian, W.; Sheng, Y.; She, B.; Jiang, Y.; Zhang, D.; Liu, Y.; Qi, J.; others Electron injection and defect passivation for high-efficiency mesoporous perovskite solar cells. *Science* **2024**, *383*, 1198–1204.

[15] Jena, A. K.; Numata, Y.; Ikegami, M.; Miyasaka, T. Role of spiro-OMeTAD in performance deterioration of perovskite solar cells at high temperature and reuse of the perovskite films to avoid Pb-waste. *Journal of Materials Chemistry A* **2018**, *6*, 2219–2230.

[16] Rombach, F. M.; Haque, S. A.; Macdonald, T. J. Lessons learned from spiro-OMeTAD and PTAA in perovskite solar cells. *Energy & Environmental Science* **2021**, *14*, 5161–5190.

[17] Wagner, L.; Suo, J.; Yang, B.; Bogachuk, D.; Gervais, E.; Pietzcker, R.; Gassmann, A.; Goldschmidt, J. C. The resource demands of multi-terawatt-scale perovskite tandem photovoltaics. *Joule* **2024**, *8*, 1142–1160.

[18] Thiesbrummel, J.; Shah, S.; Gutierrez-Partida, E.; Zu, F.; Peña-Camargo, F.; Zeiske, S.; Diekmann, J.; Ye, F.; Peters, K. P.; Brinkmann, K. O.; others Ion-induced field screening as a dominant factor in perovskite solar cell operational stability. *Nature Energy* **2024**, *9*, 664–676.

[19] Rombach, F. M.; Dasgupta, A.; Kober-Czerny, M.; Jin, H.; Ball, J. M.; Smith, J. A.; Farrar, M. D.; Snaith, H. J. Disentangling degradation pathways of narrow bandgap lead-tin perovskite material and photovoltaic devices. *Nature Communications* **2025**, *16*, 5450.

[20] Shah, S.; Yang, F.; Köhnen, E.; Ugur, E.; Khenkin, M.; Thiesbrummel, J.; Li, B.; Holte, L.; Berwig, S.; Scherler, F.; others Impact of Ion Migration on the Performance and Stability of Perovskite-Based Tandem Solar Cells. *Advanced Energy Materials* **2024**, *14*, 2400720.

[21] Balaguera, E. H.; Pra, F. J. M.; Das, C.; Torresani, L.; Bisquert, J.; Saliba, M. 'Ion-freeze' efficiency in perovskite solar cells: time scales for ion immobilization. *EES solar* **2025**, *1*, 1051–1060.

[22] Torre Cachafeiro, M. A.; Narbey, S.; Ruhstaller, B.; Nüesch, F.; Tress, W. Visualising ionic screening in perovskite solar cells: a bumpy ride along the J–V curve. *EES solar* **2025**, *1*, 762–774.

[23] Worsley, C. A.; Dunlop, T. O.; Potts, S.-J.; Garcia-Rodriguez, R.; Bolton, R. S.; Davies, M. L.; Jewell, E.; Watson, T. M. Quantifying Infiltration for Quality Control in Printed Mesoscopic Perovskite Solar Cells: A Microscopic Perspective. *ACS Applied Energy Materials* **2024**, *7*, 1938–1948.

[24] Nemnes, G. A.; Besleaga, C.; Stancu, V.; Dogaru, D. E.; Leonat, L. N.; Pintilie, L.; Torfason, K.; Ilkov, M.; Manolescu, A.; Pintilie, I. Normal and inverted hysteresis in perovskite solar cells. *The Journal of Physical Chemistry C* **2017**, *121*, 11207–11214.

[25] Bogachuk, D.; Saddedine, K.; Martineau, D.; Narbey, S.; Verma, A.; Gebhardt, P.; Herterich, J. P.; Glissmann, N.; Zouhair, S.; Markert, J.; others Perovskite photovoltaic devices with carbon-based electrodes withstanding reverse-bias voltages up to–9 V and surpassing IEC 61215: 2016 international standard. *Solar Rrl* **2022**, *6*, 2100527.
